# Estrogen receptor α promotes lung cancer cell invasion via increase of and cross‐talk with infiltrated macrophages through the CCL2/CCR2/MMP9 and CXCL12/CXCR4 signaling pathways

**DOI:** 10.1002/1878-0261.12701

**Published:** 2020-06-28

**Authors:** Miao He, Weiwei Yu, Chawnshang Chang, Hiroshi Miyamoto, Xiaohong Liu, Ke Jiang, Shuyuan Yeh

**Affiliations:** ^1^ Department of Thoracic Surgery Union Hospital Tongji Medical College of Huazhong University of Science and Technology Wuhan China; ^2^ George Whipple Lab for Cancer Research Departments of Urology and Pathology and the Wilmot Cancer Center University of Rochester Medical Center Rochester NY USA

**Keywords:** estrogen receptor α, macrophage, non‐small‐cell lung cancer

## Abstract

Data analysis of clinical samples suggests that higher estrogen receptor α (ERα) expression could be associated with worse overall survival in some patients with non‐small‐cell lung cancer (NSCLC). Immunofluorescence results further showed that higher ERα expression was linked to larger numbers of infiltrated macrophages in NSCLC tissues. However, the detailed mechanisms underlying this phenomenon remain unclear. Results from *in vitro* studies with multiple cell lines revealed that, in NSCLC cells, ERα can activate the CCL2/CCR2 axis to promote macrophage infiltration, M2 polarization, and MMP9 production, which can then increase NSCLC cell invasion. Mechanistic studies using chromatin immunoprecipitation and promoter luciferase assays demonstrated that ERα could bind to estrogen response elements (EREs) on the CCL2 promoter to increase CCL2 expression. Furthermore, ERα‐increased macrophage infiltration can induce a positive feedback mechanism to increase lung cancer cell ERα expression *via* the up‐regulation of the CXCL12/CXCR4 pathway. Targeting these newly identified pathways, NSCLC ERα‐increased macrophage infiltration or the macrophage‐to‐NSCLC CXCL12/CXCR4/ERα signal, with anti‐estrogens or CCR2/CXCR4 antagonists, may help in the development of new alternative therapies to better treat NSCLC.

## Graphical abstract

Analyses of TCGA database and our clinical samples showed that ERα correlates with worse prognosis and increased macrophage infiltration. Mechanistic study proved that ERα triggers NSCLC invasion *via* interaction with macrophages. Translational studies on mouse models proved that targeting ERα‐related pathways may provide benefits for NSCLC patients in the future.

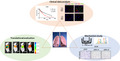

**Abbreviations**
CM: conditioned medium
ERs: estrogen receptors
ERα: estrogen receptor α
ERE: estrogen response element
IHC: immunohistochemistry
NSCLC: non‐small‐cell lung cancer
LUAD: lung adenocarcinoma
LUSC: lung squamous cell carcinoma
Mφ: macrophage
M‐CSF: macrophage colony stimulating factor
MMP: matrix metalloproteinase
MPP: methyl‐piperidino‐pyrazole
SNP: single nucleotide polymorphisms
TAM: tumor‐associated macrophage
TCGA: The Cancer Genome Atlas

## 1 Introduction

Non‐small‐cell lung cancer (NSCLC) has long been the type of cancer with the highest mortality across the world. Among various factors linked to this disease, the contributing role of estrogen and estrogen‐related pathways has also been suggested in the past decade. Direct evidence came from population studies showing that postmenopausal women live longer than men at similar ages (Albain *et al*., 1991), while hormone replacement therapy is associated with more rapid progression of the disease (Ganti *et al*., 2006; Slatore *et al*., 2010). These suggest that estrogens and estrogen receptors (ERs) play promoting roles in the progression of NSCLC.

There are two major types of ERs, ERα and ERβ. Previous work from our laboratory has proved that ERβ can promote the NSCLC *via* inducing vasculogenic mimicry and invasion in lung cancer cells (Yu *et al*., 2018). We then were interested in testing whether ERα was independently involved in the progression of NSCLC by studying the underlying mechanisms. Here, we used two specific ERα shRNAs, shERα#1 and shERα#2, together with an ERα‐specific antagonist, to demonstrate the role of ERα in NSCLC. Although there have been controversies on the role of ERα on the pathogenesis of NSCLC, the majority of literature studies using resected NSCLC samples show that expression of ERα is associated with poorer overall survival after surgery (Kawai *et al*., 2005). In accordance with the above notion, patients with higher ERα expression are less likely to gain benefits from adjuvant chemotherapy and radiation therapy (Brueckl *et al*., 2013; Rades *et al*., 2012). Regarding the possible faults due to subjective measurement of immunohistochemistry in the above studies, a study employing qPCR shows similar results that ERα mRNA levels are significantly associated with worse NSCLC prognosis (Olivo‐Marston *et al*., 2010). This study also proved a relationship between specific single nucleotide polymorphisms (SNPs) leading to high ERα expression and poor NSCLC outcomes. On the other hand, one similar study using qPCR indicated that ERα could be a good prognostic factor for metastatic NSCLC (Brueckl *et al*., 2013). However, deeper scrutiny reveals that this difference could be due to the more advanced stages of the samples included in the study.

In the case of breast cancer, ERα is a driving factor for the initiation and earlier progressive stages of the disease, while may also confer a positive response to endocrine therapy employed in more advanced stages (Busonero *et al*., 2018). For NSCLC, ERα expressed in earlier stages may boost tumor initiation, growth, and invasion, while as the disease progresses to a later stage, its role can be different. Thus, the overall evidence provided by clinical studies pointed to a promoting role of ERα in NSCLC initiation; however, the underlying mechanisms remain largely unknown. Recently, tumor‐associated macrophages (TAMs) have been proven to play an important role in NSCLC cell growth and invasion (Schmall *et al*., 2015), supporting a protumor role of macrophages in the tumor microenvironment. Interestingly, one report shows that ERα expressed in tumor cells can promote macrophage infiltration (Svensson *et al*., 2015). Thus, it is rational to hypothesize that ERα could function through regulating macrophage infiltration or functions to affect NSCLC progression.

Moreover, previous reports also show that macrophages can influence the expression of ERα in tumor cells (Ning *et al*., 2016; Stossi *et al*., 2012; Tong *et al*., 2016). While infiltrated macrophages can elicit loss of ERα expression in breast cancer cells (Stossi *et al*., 2012), ERα expression is induced by macrophages in endometrial cancer (Ning *et al*., 2016; Tong *et al*., 2016). These highlight the importance of the interaction between ERα and macrophages in the progression of tumors. Nevertheless, the effect of infiltrating macrophages on ERα expression or activity in lung cancer cells, as well as its role in NSCLC progressions, is largely unknown.

In this study, we tested our hypothesis that ERα may function *via* interaction with macrophages to trigger NSCLC invasion, as well as the possible molecular mechanisms involved, and thereafter could provide tumor‐supporting signals to stimulate progression of NSCLC. We first analyzed the online TCGA database and our clinical samples, and then applied the transwell system and molecular biology methods for phenotype and mechanistic studies. Later, animal models with tumor xenografts were used to test possible therapies targeting the related pathways. Our study may improve our understanding of the role of ERα in NSCLC and may provide some hints for future therapy.

## 2 Materials and methods

### 2.1 Cell lines and human tissue samples

Human NSCLC cell lines A549 (ATCC CCL‐185), H1299 (ATCC CRL‐580), human acute monocytic leukemia cell line THP‐1 (ATCC TIB‐202), and mouse Lewis lung carcinoma cell line LLC1 (ATCC CRL‐1642) were purchased from the American Type Culture Collection (ATCC, Rockville, MD, USA). A549 and H1299 were maintained in RPMI‐1640 media with 10% FBS and 1% penicillin/streptomycin. LLC1 was maintained in DMEM media with 10% FBS and 1% penicillin/streptomycin. THP‐1 cells were maintained in RPMI‐1640 medium with 10% heat‐inactivated FBS, 1% penicillin/streptomycin, and 2‐mercaptoethanol to a final concentration of 0.05 mm. All cultures were grown in a humidified 5% CO_2_ incubator at 37°C. Human tissue samples were provided by Department of Thoracic Surgery, Wuhan Union Hospital. All samples were collected for use in research after patients signed the Informed Consent.

### 2.2 Isolation and primary culture of macrophages from B6 mice

B6 mice were euthanized by CO_2_ asphyxiation, which was followed by cervical dislocation. After sterilization in 70% ethanol, femur bones were isolated and washed with PBS. Bones were cut at both ends, and bone marrow was flushed out by syringes with RPMI media containing 10% heat‐inactivated FBS. Then, bone marrow fluid was centrifuged at 250 ***g*** for 10 min, and cells were collected and then cultured in RPMI media containing macrophage colony‐stimulating factor (M‐CSF 20 ng·mL^−1^). With 6 days of culture, primary macrophages were mature for later experimentation.

### 2.3 Reagents and materials

The GAPDH (6C5) and β‐actin (C4) antibodies were purchased from Santa Cruz Biotechnology (Dallas, TX, USA). The anti‐human ERα (D8H8), ERK1/2 (137F5), p‐ERK1/2 (197G2), AKT (11E7), p‐AKT (244F9), and p‐STAT3 (D3A7) antibodies for western blot were purchased from Cell Signaling Technology (Boston, MA, USA); MMP‐9 (ab38898) antibody was from Abcam (Cambridge, MA, USA). The anti‐mouse ERα (E115) for western blot was from Abcam. The CXCL12 (AF‐310‐NA) antibody for western blot was from R&D Systems (Minneapolis, MN, USA). Anti‐mouse/anti‐rabbit secondary antibody for western blot was from Invitrogen (Carlsbad, CA, USA). Normal rabbit IgG was also from Santa Cruz Biotechnology. MPP (CAS 911295‐24‐4) was from Bio‐Techne Corporation (Minneapolis, MN, USA). PMA (CAS 16561‐29‐8) and CCR2 antagonist (CAS 445479‐97‐0) were from MilliporeSigma (Burlington, MA, USA). AMD3100 (CAS 155148‐31‐5) was from Bio‐Techne Corporation. U0126 (CAS 109511‐58‐2) was from Cell Signaling Technology. MK‐2206 (CAS 1032350‐13‐2) and fulvestrant (CAS 129453‐61‐8) were from Selleckchem (Houston, TX, USA).

### 2.4 Lentiviral infection

The cDNA was cloned into PmeI site of pWPI lentiviral vector, and shRNA was cloned into AgeI site of pLKO.1 lentiviral vector. The 293T packaging cells were transiently transfected with pMD2.G, psPAX2, and pWPI vector/pWPI‐cDNA, or pLKO vector/pLKO‐shRNA to produce lentiviral particles. The supernatants containing lentiviral particles were collected 48 h post‐transfection of 293T cells. The lentiviral supernatants were then filtered and used to transduce NSCLC or THP‐1 cells for 48 h.

### 2.5 Migration assay

Migration assay was carried out as was described previously (Yeh *et al*., 2016). Briefly, NSCLC cells were cultured in the bottom wells of 24‐well plates 24 h prior to the seeding of THP‐1 or primary B6 Mφ cells into the inserted transwells. To differentiate THP‐1 cells into macrophages, THP‐1 cells were treated with 50 ng·mL^−1^ PMA for 48 h. The number of migrated THP‐1 or B6 Mφ cells was examined after 48 h co‐incubation. The inserted transwells were washed with PBS and then fixed with methanol. Then, transwells were stained with 1% crystal violet (w/v, prepared in PBS) and the migrated macrophages can be shown. The THP‐1 or B6 Mφ cells that were recruited by NSCLC cells to the bottom side of the membranes were counted under microscopy, and the average numbers of six representative areas (x100 fold) were recorded.

### 2.6 Coculture experiment

To collect conditioned media (CM) and test the phenotype change of cocultured THP‐1 or B6 Mφ cells, NSCLC cells and PMA‐treated THP‐1 or primary B6 Mφ were seeded in the bottom and upper wells of 6‐well transwell plates, respectively (pore size is 0.4 μm), at the density of 1 × 10^6^/well and cultured for 48 h. Then, the CM or cells were collected for later experiments.

### 2.7 Invasion assay

CM was first collected from the 48 h coculture of NSCLC cells and THP‐1/B6 Mφ or from control NSCLC cells. NSCLC cells were seeded in the bottom wells of 6‐well plates, and macrophages were seeded in the inserted transwells (pore size is 0.4 μm). Then, CM from different coculture groups was added into new 24‐well plates, with transwells coated with Matrigel (0.2 mg·mL^−1^, 100 μL, air‐dried for 2 h) and NSCLC cells (A549, H1299, and LLC1, as in figures) seeded into each inserted transwell at the density of 5 × 10^4^/150 μL. The incubation time was 24 h, and the transwells were then washed, fixed, and stained, and invaded NSCLC cells were counted as shown in the migration assay (Yeh *et al*., 2016).

### 2.8 Immunohistochemistry and immunofluorescence

Immunohistochemistry (IHC) and immunofluorescence staining was carried out as the routine procedure by our team, which has been described previously (Chen *et al*., 2009). Briefly, the slices were first incubated with the primary antibodies, anti‐ERα (Abcam, ab93021 for IHC, E115 for immunofluorescence, Cambridge, MA, USA), anti‐CD68 (total human macrophage marker; Abcam, ab955), anti‐CD163 (M2 macrophage marker; Abcam, ab87099), or anti‐F4/80 (total mouse macrophage marker; Abcam, ab16911) in 3% BSA in PBS overnight at 4°C followed by secondary antibodies. Protein expression levels were calculated as the number of immunopositive cells × 100% divided by total number of cells/field in six randomly selected fields through microscope at 400× magnification.

### 2.9 Protein extraction and western blot

Proteins were collected through lysing of cells in RIPA buffer. Equal amounts of protein from each experimental group were loaded into SDS/PAGE gel, which were then separated and transferred onto PVDF membranes (Millipore, Billerica, MA). The membranes were then blocked with nonfat milk, incubated with appropriate dilutions of specific primary antibodies, and with HRP‐conjugated secondary antibodies. Visualization was through the ECL system (Thermo Fisher Scientific, Rochester, NY, USA).

### 2.10 RNA extraction and quantitative real‐time PCR analysis

Total RNA was extracted by TRIzol reagent (Invitrogen) according to the manufacturer’s instructions. 1 μg of RNAs from each experimental group was used for reverse transcription by qScript cDNA SuperMix (Quantabio, Beverly, MA, USA). The obtained cDNAs were applied for qPCR using a SYBR Green Bio‐Rad CFX96 system. Gene mRNA expression levels were normalized to the mRNA level of GAPDH (NSCLC cells) or β‐actin (THP‐1 or B6 Mφ cells). Primers used are listed in supplementary data (Table S1).

### 2.11 Chromatin immunoprecipitation assay (ChIP)

Cell lysates were cleared by sequential incubation with normal rabbit IgG (sc‐2027, Santa Cruz Biotechnology) and protein A‐agarose. 2.0 μg of anti‐ERα antibody was then added and incubated with the cell lysates at 4°C overnight. Equal amounts of IgG were used in the negative control group, which was incubated under the same condition. The PCR was done, and products were identified by agarose gel electrophoresis.

### 2.12 CCL2 promoter luciferase assay

The promoter luciferase assay was done as described previously (Yu *et al*., 2018). The human promoter region of CCL2 was constructed into pGL3‐basic vector (Promega, Madison, WI, USA). Cells were plated in 24‐well plates, and the cDNAs were transfected using Lipofectamine (Invitrogen) according to the manufacturer's instructions. The pRL‐TK was used as internal control. Luciferase activity was measured by Dual‐Luciferase Assay (Promega) according to the manufacturer's instruction.

### 2.13 Mouse model of orthotopic tumor implantation and drug administration

Eight‐week‐old female nude mice were purchased from NCI. Logarithmically growing A549 cells were transduced with luciferase and with/without lentiviral ERα (oeERα). Lung cancer cells (2 × 10^6^) were suspended in 50 μL Matrigel (Becton Dickinson, CA, USA) and injected into the left lateral thorax of the mice as described previously (Yu *et al*., 2018). After 7 days of tumor development, mice were randomized into five groups and treated every other day by intrathoracic injection. The five groups were (1) pWPI‐A549 cells with DMSO, (2) oeERα‐A549 cells with DMSO, (3) oeERα‐A549 cells with CCR2 antagonist (50 μg·kg^−1^), (4) oeERα‐A549 cells with AMD3100 (5 mg·kg^−1^), and (5) oeERα‐A549 cells with fulvestrant (5 mg·kg^−1^). Tumor development was monitored by Fluorescent Image (IVIS Spectrum, Caliper Life Science, Hopkinton, MA, USA). After 28 days of treatments, the mice were imaged by IVIS and sacrificed, and tumors were collected, weighed, and prepared for further analysis. We performed the experiment with eight mice per group of treatment. In each group, the mice with the highest and the lowest luciferase signals were removed to eliminate any outliers. In order to confirm the results, we replicated the animal experiment 3 independent times. All animal experiments were performed in accordance with the guidelines of the University of Rochester Medical Center Animal Care and Use Committee for animal experiments.

### 2.14 Survival analysis for NSCLC patients

Survival data for NSCLC patients based on ERα mRNA expression were obtained from the TCGA database. The TCGA project collected gene expression information together with general clinical information including age, gender, and tumor staging. The cutoff point to differentiate high and low expression is determined by median ERα mRNA level. Survival data for NSCLC patients based on ERα protein expression were retrieved from medical records of Wuhan Union Hospital. The TCGA program has made sure that every contributing clinical site verified that IRB approval has been received, and the ethical policies are posted on the project website (https://www.cancer.gov/about‐nci/organization/ccg/research/structural‐genomics/tcga/history/policies). Also, all patients enrolled in the Union Hospital cohort provided Informed Consent. Patient information and tissue samples were collected according to the ethical guidelines set by the Internal Review Board of Wuhan Union Hospital. General characteristics of the patients analyzed are also shown in Tables S2 and S3. The study methodologies on human patients also conformed to the standards set by the Declaration of Helsinki.

### 2.15 Statistical methods

All experiments were performed in triplicate and at least three times. Data were presented as mean ± SD. Statistical analyses involved were carried out using Student’s *t*‐test, and log‐rank (Mantel–Cox) test with SPSS 22 (IBM Corp, Armonk, NY, USA) or graphpad prism 6 (GraphPad Software, La Jolla, CA, USA). *P* < 0.05 was considered statistically significant.

## 3 Results

### 3.1 ERα is correlated with a worse prognosis and increased macrophage infiltration in the early‐stage NSCLC patients

**Fig. 1:**
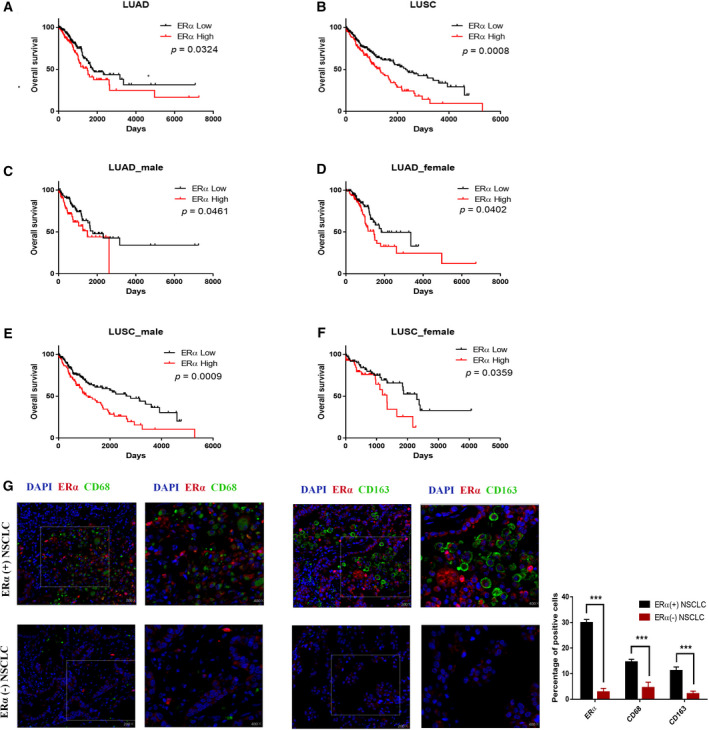
Estrogen receptor α is correlated with worse prognosis and macrophage infiltration in early‐stage NSCLC patients. (A,B) Survival curve based on TCGA database indicated that high levels of ERα mRNA are associated with shorter overall survival in both LUAD and LUSC. (C–F) Differential analysis based on gender showed that ERα mRNA is associated with poorer overall survival both in male (C, E) and in female (D, F) NSCLC patients. (G) Immunofluorescence assay comparing ERα, CD68, and CD163 in human ERα‐positive and ERα‐negative NSCLC samples. Nuclei (blue), ERα (red), CD68 (green), and CD163 (green) were shown. 200× magnification was shown for the left image, and 400× magnification was shown for the right image. Log‐rank (Mantel–Cox) test was applied to compare survival data in Fig. 1A–F, and Student’s t‐test was used to compare the means between groups in Fig. 1G. Experiments were done at least in 3 replicates. Results were presented as mean ± SD, ***P < 0.001.

We first performed the human clinical survey via analyzing the TCGA database for the association between ERα expression and the overall survival of NSCLC patients. The results revealed that for both lung adenocarcinoma (LUAD) and lung squamous cell carcinoma (LUSC), there is a significant linkage between ERα mRNA expression and worse overall survival in the early‐stage patients (TNM stage IA‐IIB) (Fig. 1A,B). As estrogen levels are higher in female patients, we are interested to see whether the significant linkage is impacted by the gender difference. The results from multiple analyses revealed that higher ERα is a worse prognostic factor in LUAD (Fig. 1C,D) and LUSC (Fig. 1E,F) from both male and female NSCLC patients. Also, we analyzed the association between ERα protein expression and overall survival through immunohistochemistry method in a patient cohort from Wuhan Union hospital, in which ERα‐negative and ERα‐positive patients are 1 : 1 matched (Fig. S1A‐B, Table. S3). Similar to the results obtained from TCGA database, ERα protein expression also correlates with significantly worse overall survival in this Wuhan Union Hospital cohort. Together, results from human clinical surveys (Figs. 1A–F, S1A–B) suggest that higher activation or expression of ERα may lead to worse prognosis in both male and female NSCLC patients.

**Fig. 2:**
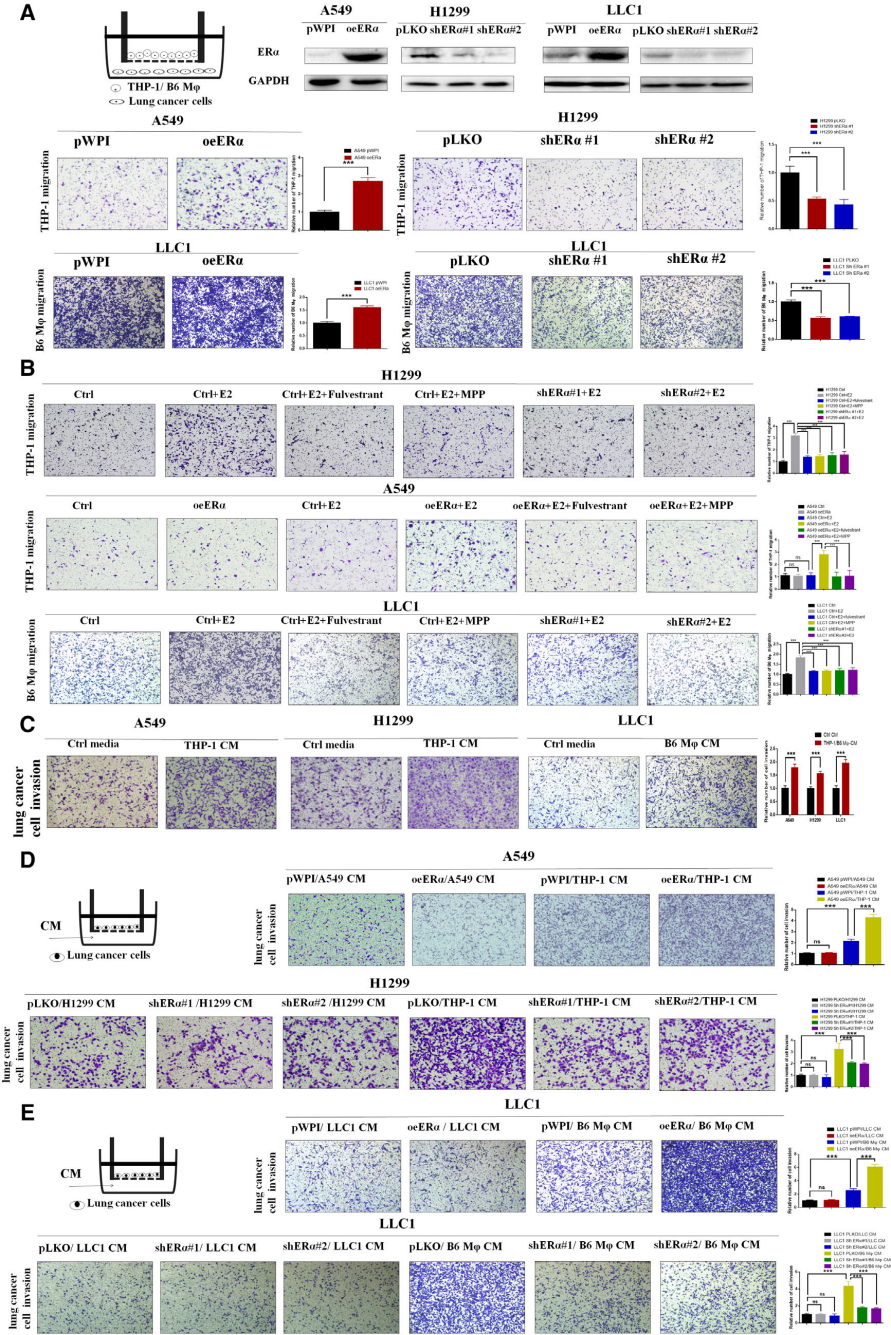
E2/ERα signals in lung cancer cells could promote the macrophage recruitment to induce cancer invasion. (A) The carton (left) illustrates macrophage migration systems. PMA‐activated THP‐1 or primary B6 Mφ cells were seeded in the upper chambers, and lung cancer cells were seeded in the lower chambers of the 24‐well transwell migration systems. Migrated THP‐1 cells/B6 Mφ were checked after 48 h incubation. (B) E2 pretreated ERα‐positive cells can promote the macrophage recruitment. H1299 (vector or shERα #1, #2), A549 (vector or oeERα), or LLC1 (vector, or shERα #1, or shERα #2) cultured in charcoal‐stripped FBS media were treated with control and 10 nm E2, with/without 1 μm MPP, with/without 10 μm fulvestrant for 48 h, and then seeded in the lower chamber of transwell migration systems for checking the ability to recruit THP‐1 cells or B6 Mφ, respectively. (C) CMs were collected from coculture of lung cancer cells with/without THP‐1 or B6 Mφ cells (4 : 1) for 48 h. The CMs were then added to the bottom well of 24‐well plates. Lung cancer cells were seeded into inserted transwells precoated with Matrigel. After 24 h incubation, invaded lung cancer cells were counted and compared. (D) CMs were collected from coculture of A549 (vector or oeERα) or H1299 (vector, or shERα #1, or shERα #2) cells with PMA‐activated THP‐1 cells. The CMs were then added in the lower chambers of transwells to test the effect on lung cancer cell (A549 and H1299) invasion. (E) CMs were collected from coculture of LLC1 (vector or oeERα) or LLC1 (vector, or shERα #1, or shERα #2) cells with primary B6 Mφ cells. The CMs were then added in the lower chambers of transwells to test the effect on lung cancer cell (LLC1) invasion. 100 × magnification of images was shown for the migration and invasion assay. Student’s t‐test was used to analyze data in Fig. 2A–E. Experiments were done at least in 3 replicates. Results were presented as mean ± SD, **P < 0.01, ***P < 0.001. ns, not significant.

To explore the underlying mechanisms why ERα is associated with a worse prognosis of NSCLC, we first performed immunofluorescence assay and IHC staining to test the subcellular location of ERα, which shows that ERα can be mainly stained in the nuclei with relatively weak cytosol staining in NSCLC cells (Figs. S1B, S2). We then performed MTT growth assay and Matrigel invasion assay on the lung A549 (vector or oeERα) cells cultured in regular media with 10% FBS and H1299 cells cultured in charcoal‐stripped FBS media treated with mock, 17β‐estradiol (E2), and with ER antagonist fulvestrant or specific ERα antagonist MPP. The results showed that overexpression of ERα, treatment with agonist (E2) and/or antagonists (fulvestrant and MPP), does not have significant effects on cell growth or invasion (Fig. S3A–D). Also, the expression levels of ERα in A549 and H1299 cells are shown in Fig. S4A, which indicates that A549 cells have relatively lower, while H1299 cells have relatively higher, endogenous expression of ERα. The expression of ERα in A549 (vector or oeERα) is also shown in Fig. 2A.

As recent studies indicated that infiltrated macrophages could affect the tumor progression, we then performed the immunofluorescence staining of 20 human NSCLC samples with antibodies to ERα and macrophage markers (CD68 and CD163) to examine their relationship in the microenvironment. The results from immunofluorescence staining indicated that higher ERα is linked to higher level of infiltrated macrophages in the tumor microenvironment (Fig. 1G).

Together, results from Fig. 1A–G and Figs. S1–S4 suggest that ERα may increase NSCLC progression via increasing the number of infiltrated tumor‐associated macrophages.

### 3.2 Mechanism dissection of why a higher ERα expression in lung cancer cells could promote the macrophage infiltration

Macrophages have been linked to the promotion of cancer cell invasion (Joyce and Pollard, 2009; Li *et al*., 2017), and our above human clinical sample survey also linked ERα to the infiltrating macrophages. Therefore, we applied the transwell migration assay with NSCLC cells seeded in the lower chamber and PMA‐treated THP‐1 or primary B6 macrophages (Mφ) seeded in the upper wells (Fig. 2A) to examine the impact of NSCLC ERα expression on the recruitment of macrophages. Our results revealed that increasing ERα in A549 cells via ectopic transfection of ERα‐cDNA led to an increased THP‐1 recruitment (Fig. 2A, middle left). In contrast, decreasing ERα in H1299 cells via adding 2 separate ERα‐shRNAs led to a reduced macrophage recruitment (Fig. 2A middle right). Similar results were also gained when we replaced THP‐1 cells with B6 Mφ. Increasing ERα in LLC1 cells via ectopic transfection of ERα‐cDNA led to an increased B6 Mφ recruitment (Fig. 2A, lower left), and decreasing ERα in LLC1 cells via adding 2 different ERα‐shRNAs led to a reduced B6 Mφ recruitment (Fig. 2A lower right). To further validate the involvement of ERα activation in the attraction of macrophages, we cultured ERα‐negative cells (A549, vector or oeERα) and ERα‐positive cells (H1299 and LLC, vector or shERα) in charcoal‐stripped FBS media with or without 17β‐estradiol (E2) and treated the cells with or without antagonist, fulvestrant or MPP (Harrington *et al*., 2003) (Fig. 2B). The results proved that ligand activation of ERα is necessary for its role in promoting macrophage recruitment by NSCLC cells.

We then applied the Matrigel transwell assay to compare the effect of control and THP‐1/B6 Mφ‐CM on lung cancer cell invasion (Fig. 2C). Results showed that THP‐1 CM indeed could significantly increase A549 and H1299 invasion compared with control CM, and B6 Mφ CM could significantly increase LLC1 invasion compared with control CM. Furthermore, we collected CM from coculture of THP‐1 with lung cancer A549 cells (vector or oeERα) or H1299 cells (vector or shERα), and the results revealed that CM from coculture of macrophages with lung cancer A549/oeERα had better capacity to increase the lung cancer cell invasion (Fig. 2D, upper panel), and CM from coculture of macrophages with lung cancer H1299/ shERα had reduced capacity to stimulate the lung cancer cell invasion (Fig. 2D, lower panel). Similar results were also obtained when we replaced THP‐1 cells with primary B6 Mφ and human lung cancer cells with mouse LLC1 cells (Fig. 2E).

Together, results from Fig. 2A–E suggest that ERα may increase lung cancer cell invasion via increasing the macrophage recruitment.

### 3.3 Mechanism dissection of how ERα could increase infiltrated macrophages‐enhanced lung cancer cell invasion: via promoting the M2 polarization and MMP9 production of macrophages

**Fig. 3:**
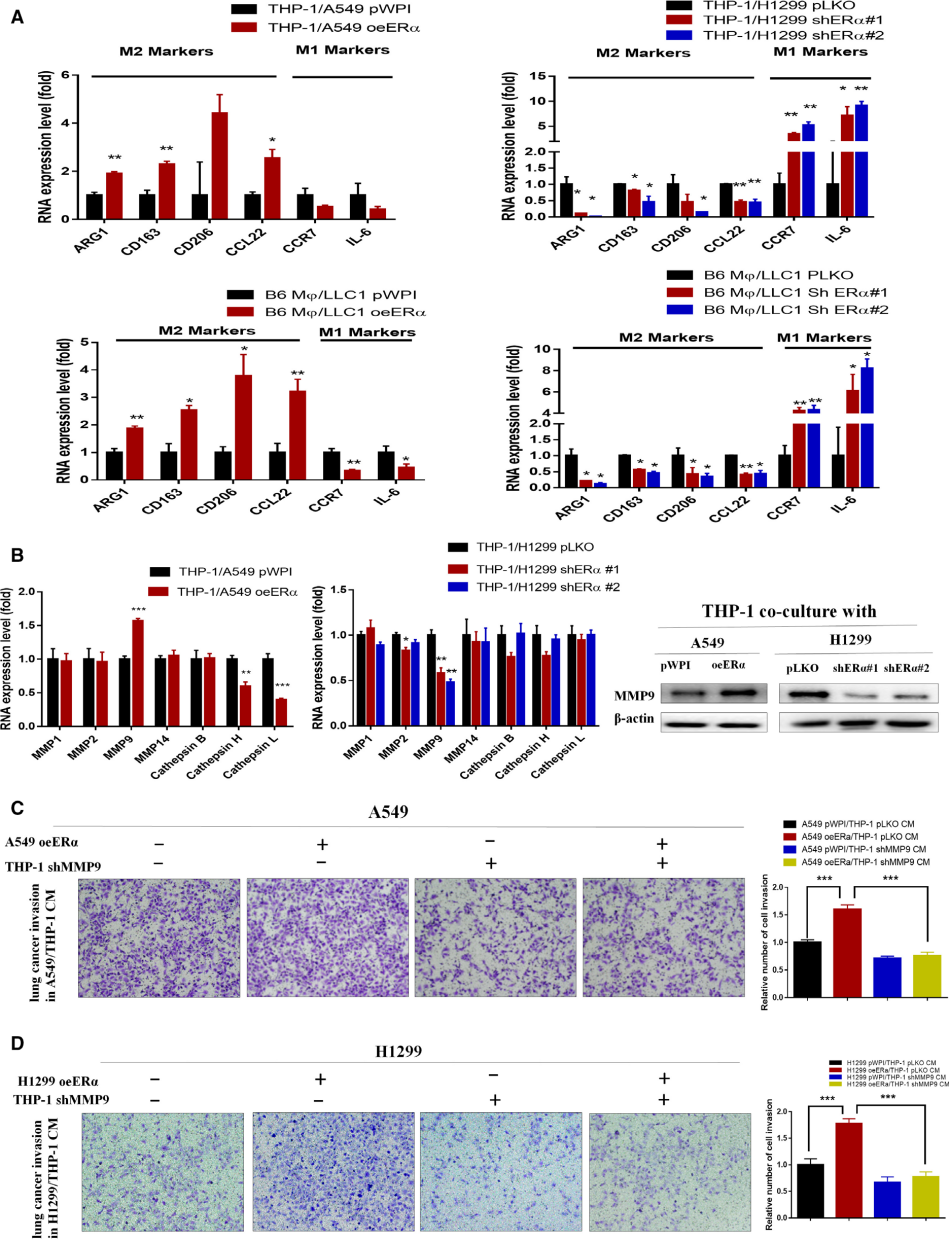
Estrogen receptor α in lung cancer cells can promote the M2 polarization and macrophage MMP9 production to facilitate cancer cell invasion. (A) THP‐1 cells were collected after coculture with A549 (upper left, vector or oeERα) and H1299 (upper right, vector or shERα) human lung cancer cells, and B6 Mφ cells were collected after coculture with LLC1 (lower left, vector or oeERα) and LLC1 (lower right, vector or shERα) mouse lung cancer cells, in order to test the change of macrophage polarization markers. (B) A group of proteases reported to promote cancer cell invasion were tested on THP‐1 cells for mRNA expression change after they were cocultured with A549 (left, vector or oeERα) and H1299 (middle, vector or shERα) by qPCR. Western blot was done to confirm the protein expression change (right). (C,D) CMs were collected from THP‐1 cells (vector or shMMP9) cocultured with (C) A549 (vector or oeERα) and (D) H1299 (vector or oeERα) to test the effect on lung cancer cell invasion. 100× magnification was shown for the images in the invasion assay. Student’s t‐test was used to compare the means between groups in A–D. Experiments were done in at least 3 replicates. Results were presented as mean ± SD, *P < 0.05, **P < 0.01, ***P < 0.001.

Next, to study the mechanism of how ERα could increase infiltrated macrophages‐enhanced lung cancer cell invasion, we first focused on the M1 to M2 polarization of macrophages, as early studies indicated this polarization is associated with tumor progression (Murray, 2017; Shapouri‐Moghaddam *et al*., 2018). We then examined the change of polarization markers by qPCR detection of the M2 markers, including ARG1, CD163, CD206, and CCL22. After coculture with lung cancer cells, the qPCR results revealed that a higher ERα in lung cancer cells could indeed increase the M2 polarization of macrophages, in both the THP‐1 cells collected from coculture with human NSCLC cells (Fig. 3A, upper left) and primary B6 Mφ collected from coculture with mouse LLC1 cells (Fig. 3A, lower left). In contrast, reducing ERα in lung cancer cells can result in a M1 polarization of cocultured THP‐1 cells or primary B6 Mφ (Fig. 3A, upper right and lower right).

As previous literature indicated that tumor‐associated macrophages (TAMs) are potent producers of many proteases, including matrix metalloproteinases (MMPs) and cathepsins (Noy and Pollard, 2014; Qian and Pollard, 2010), we then examined several protease genes, including MMP1, 2, 9, and 14 and cathepsin B, H, and L, that are related to the lung cancer cell invasion (Bröker *et al*., 2004; Merchant *et al*., 2017; Okudela *et al*., 2016; Schweiger *et al*., 2000). Our data showed that increasing ERα via adding ERα‐cDNA in lung cancer A549 cells could increase the MMP9 mRNA expression in cocultured THP‐1 cells (Fig. 3B, left). In contrast, decreasing ERα via adding lentiviral shERα #1 and shERα #2 in lung cancer H1299 cells can reduce the MMP9 mRNA expression in cocultured THP‐1 cells (Fig. 3B, middle). Similar results were also obtained when we replaced qPCR‐mRNA assay with western blot assay to test MMP9 expression at protein levels (Fig. 3B, right).

Importantly, the interruption approach using shRNA to suppress the MMP9 expression could reverse the ERα‐increased lung cancer cell invasion in A549 (Fig. 3C) and H1299 cells (Fig. 3D) [see the altered protein expression via western blot analysis in Fig. 2A and Fig. S4B‐C]. Together, results from Fig. 3A–D suggest that ERα may increase lung cancer cell invasion via increasing M2 polarization and MMP9 production by macrophages.

### 3.4 Mechanism dissection of how NSCLC ERα can increase infiltrating M2 macrophages with higher MMP9 expression: via production of CCL2

**Fig. 4:**
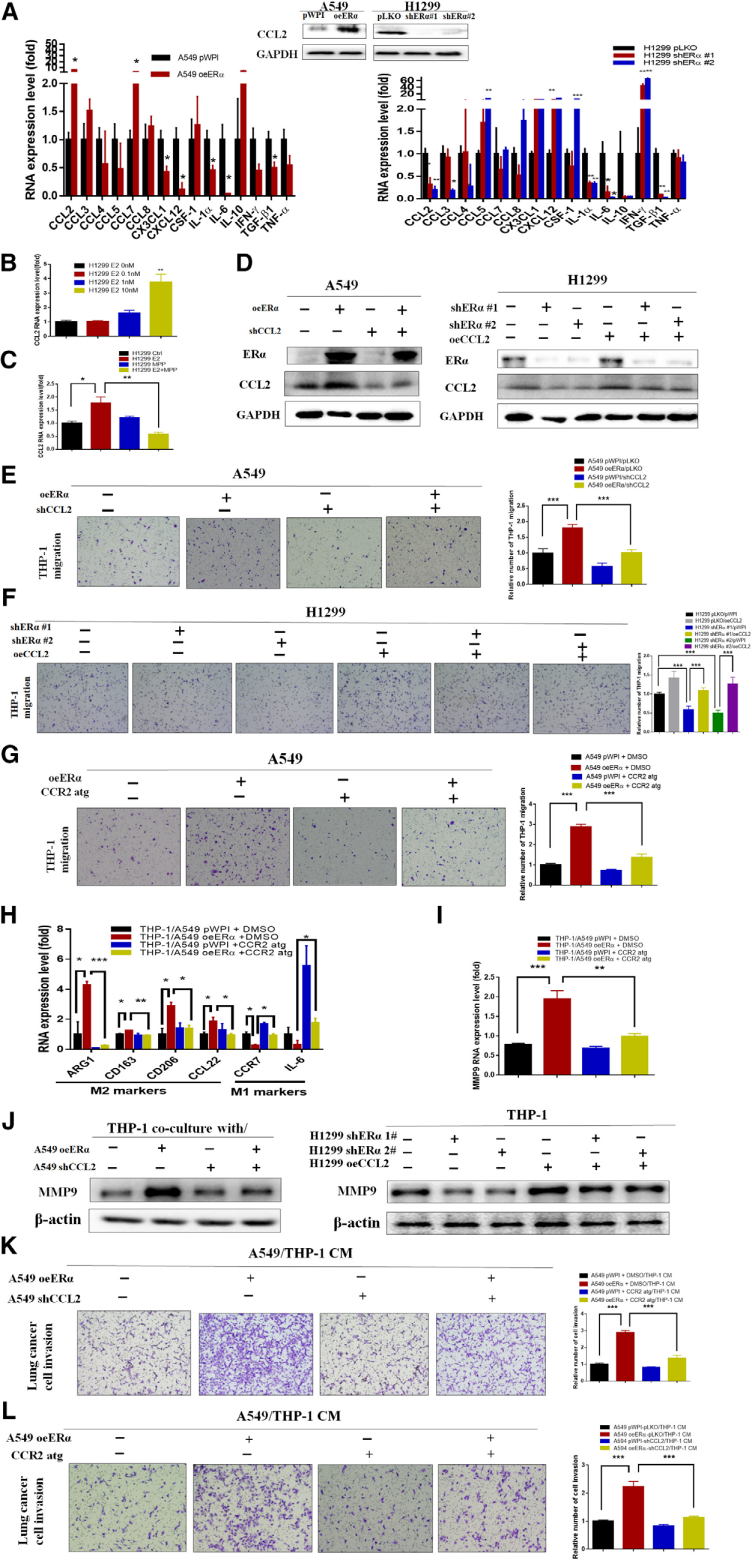
Estrogen receptor α in lung cancer cells could increase CCL2 expression to promote infiltration and MMP9 production of macrophages. (A) A group of chemokines and cytokines related to macrophage recruitment and polarization were tested by qPCR through overexpression and knockdown of ERα in A549 and H1299, respectively (left and middle). Western blots confirm that CCL2 expression is correlated with ERα expression (right). (B) Dose‐dependent increase of CCL2 mRNA levels in H1299 cells with serial doses of E2 treatments for 24h. (C) CCL2 mRNA levels were checked in H1299 cells treated with vehicle or E2 (10 nm), and with/without MPP (1 μm), for 24 h. (D) Western blot assays confirmed the efficiency of knockdown and overexpression of CCL2 in A549 (vector or oeERα) and H1299 (vector, or shERα#1, or shERα#2), respectively. (E,F) CCL2 was knocked down or overexpressed in A549 (vector or oeERα, E) or H1299 (vector, or shERα#1, or shERα#2, F) cells to test the effect on PMA‐treated THP‐1 infiltration. (G) A549 (vector or oeERα) cells were cultured in the lower wells and incubated with 25 nm CCR2 antagonist or DMSO. After 24 h, PMA‐treated THP‐1 cells were added to the upper wells for 48 h. Both the top and bottom wells contained 25 nm CCR2 antagonist. The migrated THP‐1 cells were counted and compared. (H,I) THP‐1 cells were cocultured with A549 (vector or oeERα) +/− CCR2 antagonist for 48 h to test the change of mRNA level change of macrophage polarization markers (H), and MMP9 (I). (J) Western blot assays to detect the THP‐1 MMP9 production after cocultured with A549 (vector or oeERα) with or without CCL2‐shRNA or with H1299 (vector or shERα #1, #2) with or without CCL2‐cDNA. (K,L) CMs were collected from THP‐1 cells cocultured with A549 (vector or oeERα) cells +/− CCL2‐shRNA (K), or +/− CCR2 antagonist (L). After 48 h coculture or treatment(s), CMs were collected to test their effects on A549 cell invasion. The images with 100 × magnification were shown for the migration and invasion assay. Student’s t‐test was used to analyze data in A–C, E–I, K–L. Experiments were done in at least 3 replicates. Results were presented as mean ± SD, *P < 0.05. **P < 0.01. ***P < 0.001.

To dissect the mechanism of how ERα in the lung cancer cells increases the infiltrating M2 macrophages with higher MMP9 production, we then examined those cytokines that have been linked to the macrophage recruitment and polarization (Ao *et al*., 2017; Izumi *et al*., 2013; Lee *et al*., 2013; Murdoch *et al*., 2004; Nagarsheth *et al*., 2017; Sánchez‐Martín *et al*., 2011; Su *et al*., 2014; Wang *et al*., 2013; Yeh *et al*., 2016). The results revealed that altering ERα expression via either adding ERα‐cDNA or 2 separate ERα‐shRNAs in A549 and H1299 cells, respectively, could lead to altered expression of CCL2 at the mRNA (Fig. 4A, left) and protein levels (Fig. 4A, right). Moreover, treating with E2 also led to increase the CCL2 mRNA expression in a dose‐dependent manner (Fig. 4B), and these effects could be blocked after adding an ERα‐specific antagonist MPP in H1299 (Fig. 4C).

Importantly, results from the interruption approach via adding CCL2‐shRNA or CCL2‐cDNA (see western blot analysis in Fig. 4D) can lead to reverse ERα‐cDNA‐ or ERα‐shRNA‐mediated effects on the THP‐1 recruitment by the A549 cells (Fig. 4E) and H1299 cells (Fig. 4F), respectively. Similar interruption effects were also observed when we replaced the CCL2‐shRNA with C₂₈H₃₄F₃N₅O₄S (CAS 445479‐97‐0), a CCR2‐specific antagonist (Cherney *et al*., 2008; Ding *et al*., 2015; Izumi *et al*., 2013) in A549 cells (Fig. 4G).

We further tested whether CCL2 could also play a role in this ERα‐increased M2 macrophage polarization, and qPCR results revealed that treatment with CCR2 antagonist in the coculture system can partially block the ERα‐induced M2 polarization (Fig. 4H), as well as ERα‐increased MMP9 expression in the THP‐1 cells (Fig. 4I).

We then applied western blot assay to further prove that the ERα/CCL2 axis in lung cancer cells can affect MMP9 production, and results showed that adding CCL2‐shRNA or CCL2‐cDNA (see western blot analysis in Fig. 4D) can lead to reverse the effect on production of MMP9 from THP‐1 cells cocultured with A549 cells (Fig. 4J left) or H1299 cells (Fig. 4J, right). Furthermore, A549 (vector or oeERα) cells were transduced with CCL2‐shRNA or treated with CCR2 antagonist in coculture system to collect CM (as shown by carton in Fig. 3A). Transwell invasion assay results show that CCL2‐shRNA in A549 cells and CCR2 antagonist in coculture system can partly reverse the ERα‐increased A549 cell invasion (Fig. 4K,L).

Together, results from Fig. 4A–L suggest that ERα can function via increasing CCL2/CCR2 signaling to alter the infiltrating M2 macrophages with higher MMP9 expression to increase lung cancer cell invasion.

### 3.5 Mechanism dissection of how ERα can increase CCL2 expression: via altering the transcriptional regulation

**Fig. 5:**
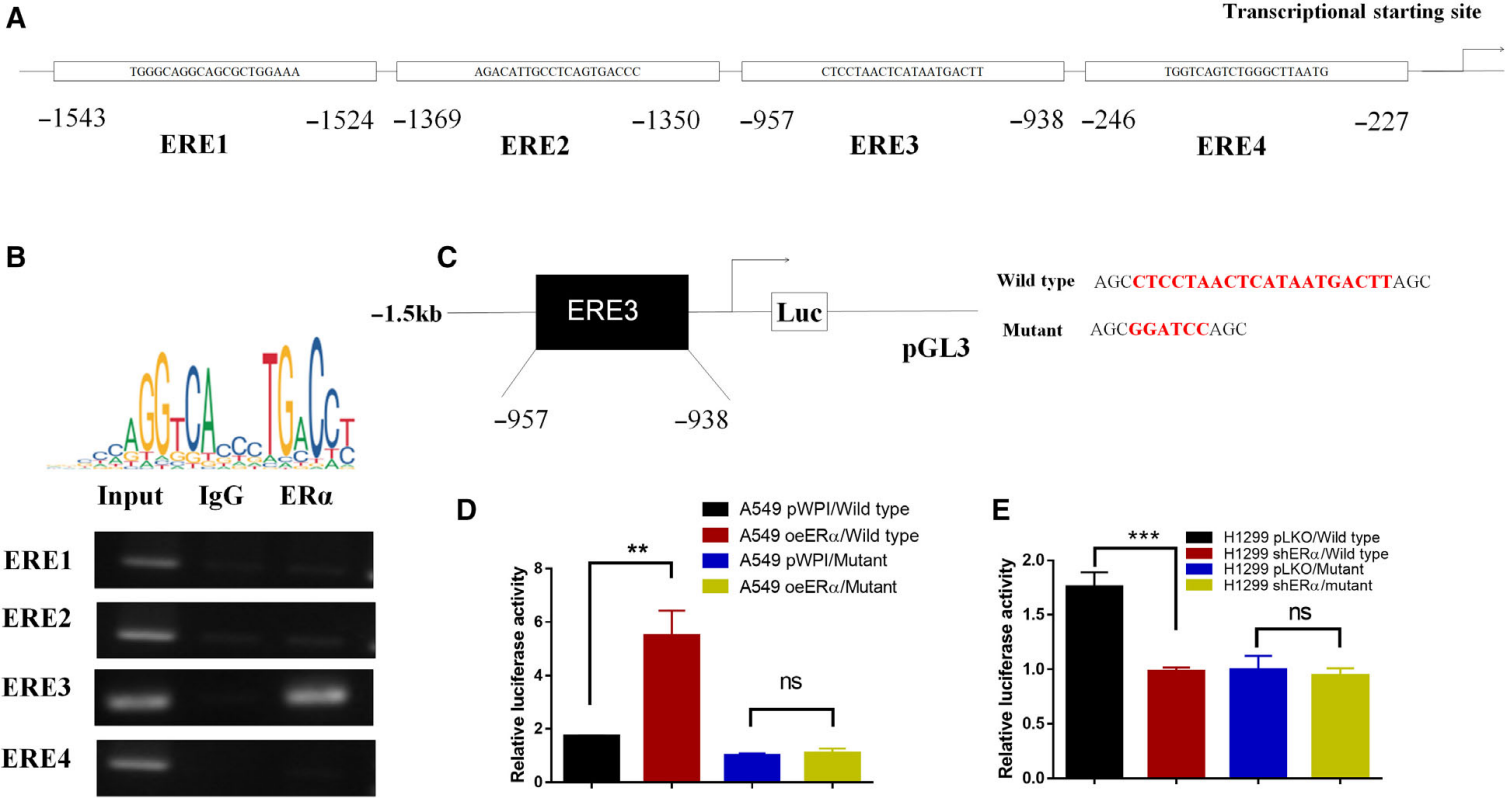
Estrogen receptor α transcriptionally regulates CCL2 production in lung cancer cells. (A) Illustration of the potential ERE sites on the CCL2 promoter region. (B) ChIP assays were performed to show ERα binding on the proposed ERE3 on CCL2 promoter region. (C) Luciferase reporter assays were used to determine whether ERα transcriptionally regulates the CCL2 promoter. (D,E) Wild‐type or mutant CCL2 promoter (ERE3)‐luciferase reporter was transfected into A549 with vector or oeERα (D), or into H1299 with vector or shERα (E). CCL2 promoter reporter activity was analyzed using the Dual‐Luciferase Assay. Experiments were done at least in 3 replicates. Results were presented as mean ± SD, and P values were calculated by Student’s t‐test. *P < 0.05, **P < 0.01, ***P < 0.001. ns, not significant.

Since ERα can increase CCL2 expression at both protein and mRNA levels (Fig. 4A), we then applied the ChIP *in vivo* binding assay to examine whether ERα can alter CCL2 expression via transcriptional regulation. The results revealed that ERα could transcriptionally regulate CCL2 expression via binding to the estrogen response element 3 (ERE3) on the promoter region (Fig. 5A,B). Furthermore, results from luciferase assay with pGL3 reporter plasmids containing the wild‐type or mutant ERE3 (Fig. 5C) revealed that increasing or decreasing the ERα expression in A549 (Fig. 5D) or H1299 (Fig. 5E) cells could lead to increased or decreased luciferase activity for the pGL3 reporter plasmids containing the wild‐type, but not mutant ERE3 (Fig. 5C–E).

Together, results from Fig. 5A–E suggest that ERα can increase macrophage infiltration via transcriptional regulation of the cytokine CCL2 expression in the lung cancer cells.

### 3.6 ERα‐increased infiltrated macrophages can up‐regulate ERα expression in lung cancer cells via a positive feedback pattern

**Fig. 6:**
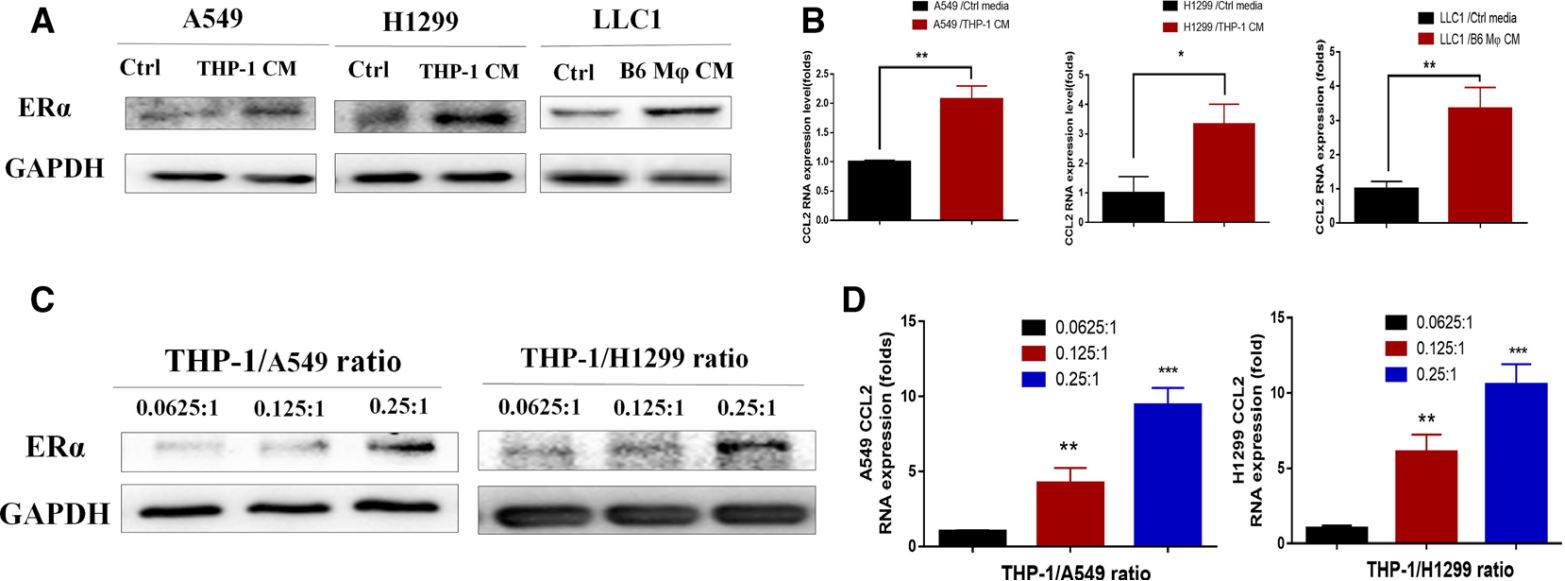
Infiltrated macrophages induce the expressions of ERα and its downstream gene CCL2 in lung cancer cells. (A,B) A549 and H1299 cells were cultured in control media or THP‐1 conditioned media for 48 h, and LLC1 cells were cultured in control media or primary B6 Mφ‐conditioned media for 48 h to test the ERα expression by western blots (A), as well as CCL2 mRNA by qPCR (B). (C,D) We collected lung cancer cells from coculture of increasing ratio of THP‐1 cells for 48 h and tested the expression of ERα in lung cancer cells by western blots (C) and CCL2 mRNA expression by qPCR (D). Student’s *t*‐test was used to analyze data in (B) and (D). Experiments were done at least in 3 replicates. Results were presented as mean ± SD, *P < 0.05, **P < 0.01, ***P < 0.001.

To explore the feedback effect of increased infiltrating macrophages on the expression of ERα in lung cancer cells, we cultured the cells with control or THP‐1/B6 Mφ CM. The results from western blots revealed that CM from coculture of THP‐1 cells or primary B6 Mφ cells with lung cancer cells could increase the expression of ERα (Fig. 6A), and CCL2 (Fig. 6B), in lung cancer A549, H1299, and LLC1 cells. Moreover, increasing numbers of THP‐1 cells within the coculture system could also lead to increase the expression of ERα (Fig. 6C) and CCL2 (Fig. 6D) in the lung cancer cells in a dose‐dependent manner. Together, results from Fig. 6A–D suggest that ERα‐increased infiltrating macrophages may function via a positive feedback mechanism to increase the ERα expression.

### 3.7 Mechanism dissection of how increasing infiltrated macrophages can function via feedback mechanism to increase ERα in lung cancer cells: via CXCL12 expression

**Fig. 7:**
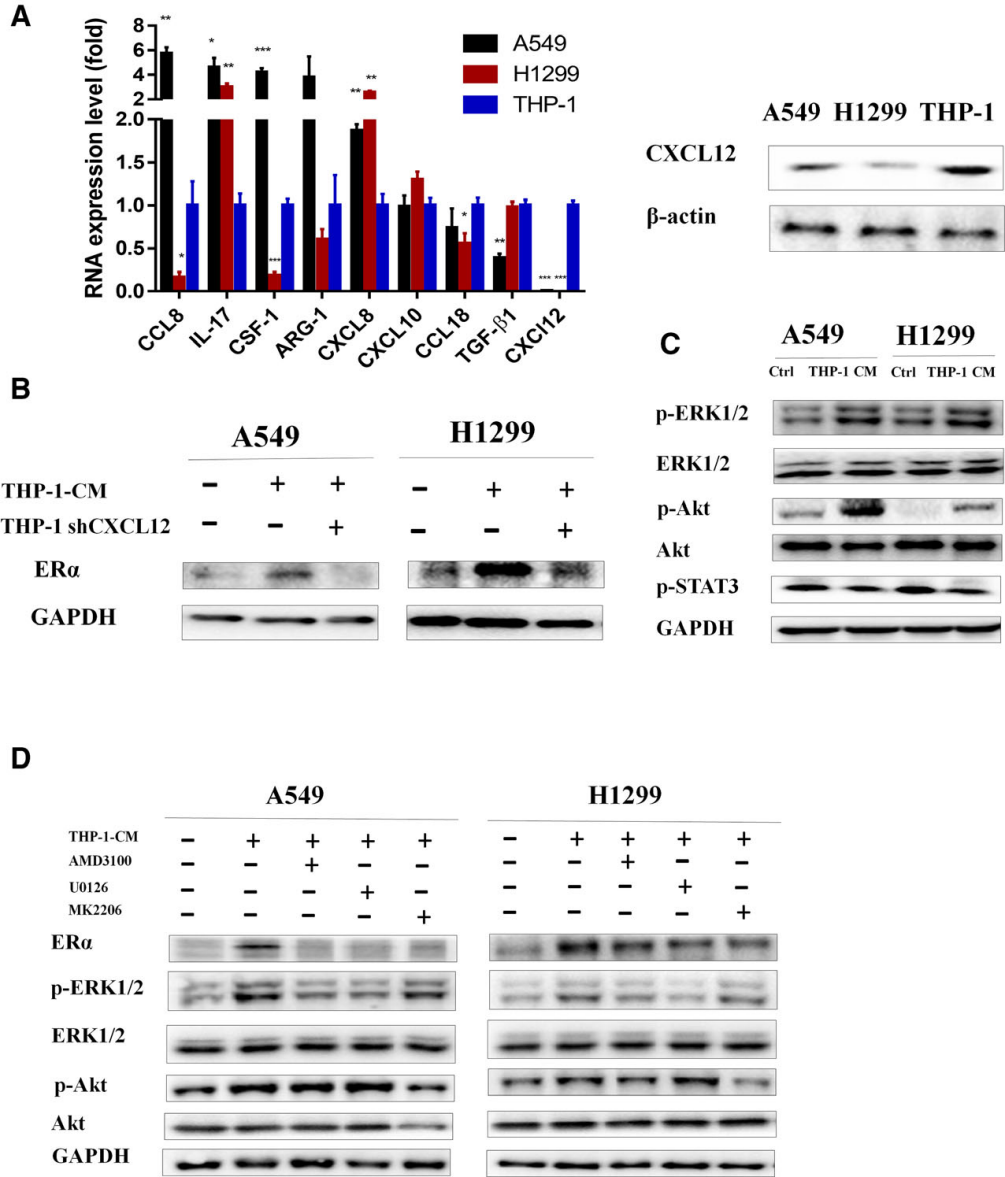
Infiltrated macrophages induce ERα expression in lung cancer cells through CXCL12. (A) We tested some cytokines that have been reported to increase ERα expression between lung cancer cells and macrophages (left). Western blot was done to confirm the protein expression change (right). (B) We collected THP‐1 (vehicle or shCXCL12)‐CM to culture lung cancer cells for 48 h and tested the change of effect on ERα expression. (C) Downstream pathway of CXCL12 was tested in A549 and H1299 cells after coculture in THP‐1‐CM for 48 h. (D) Specific antagonist of CXCR4 (AMD3100, 1µM), ERK phosphorylation (U0126, 10 μm), and Akt phosphorylation (MK2206, 1 µm) was added during culture of lung cancer cells, and the effect of ERα expression, as well as ERK phosphorylation and Akt phosphorylation, was tested by western blot. Experiments were done at least in 3 replicates. Student’s t‐test was used to analyze data in 7A. Results were presented as mean ± SD, *P < 0.05, **P < 0.01, ***P < 0.001.

To further dissect the mechanism of how infiltrating macrophages can increase the ERα expression in the lung cancer cells, we then examined those reported cytokines that may affect ERα expression (Farmaki *et al*., 2016; Katz *et al*., 2013; Kirma *et al*., 2004; Lazennec and Richmond, 2010; Levano *et al*., 2011; Ning *et al*., 2016; Perez *et al*., 2012; Sakumoto *et al*., 2017; Sauvé *et al*., 2009). The results revealed that THP‐1 cells might express higher CXCL12 compared with lung cancer A549 and H1299 cells at mRNA levels (Fig. 7A, left), which is also confirmed through western blot on protein level (Fig. 7B, right). Moreover, adding CXCL12‐shRNA into THP‐1 cells within the coculture system can also lead to reversing the infiltrated macrophages‐increased ERα expression in A549 and H1299 lung cancer cells (Fig. 7B) (see the altered CXCL12 expression in THP‐1 cells via western blot analysis in the Fig. S4D).

CXCR4 is the primary binding receptor of CXCL12 (Teicher and Fricker, 2010). We then tested the change of major downstream pathways of CXCR4 while being acted on by CXCL12, to identify which pathways of CXCR4 may be involved in the induction of ERα in NSCLC cells. Western blot data reveal that THP‐1‐CM‐cultured A549 and H1299 show higher expression of phosphorylated ERK and Akt, while no change of phosphorylated STAT3 (Fig. 7C). Then, inhibitor of ERK phosphorylation (U0126, 10 μm), inhibitor of Akt phosphorylation (MK‐2206, 1 µM) (Zhang *et al*., 2015), as well as the specific antagonist of CXCR4 (AMD3100, 1 µM) (Hatse *et al*., 2002), were added into THP‐1‐CM, and results show that they can all efficiently reverse CM‐increased ERα expression in the lung cancer cells (Fig. 7D). These results suggest that the ERK and Akt pathways following the activation of CXCR4 by CXCL12 may function in the increasing expression of ERα in NSCLC cells during coculture with macrophages.

Together, results from Fig. 7A–D suggest that the induction of ERα expression in the lung cancer cells by infiltrated macrophages is through the CXCL2/CXCR4 axis, which may also involve the downstream ERK and Akt pathways.

### 3.8 Preclinical study using *in vivo* mouse model with xenografted A549 cells

To confirm the above *in vitro* cell line data, we used an *in vivo* experimental lung mouse cancer model, and A549 cells were into the left thorax of nude mice. A549 cells were first stably transfected with luciferase reporter gene and then prepared with or without oeERα. One week after tumor injection, the mice were randomly assigned into five groups for treatment as follows. (1) pWPI‐A549 cells with DMSO, (2) oeERα‐A549 cells with DMSO, (3) oeERα‐A549 cells with CCR2 antagonist (50 μg·kg^−1^), (4) oeERα‐A549 cells with AMD3100 (5 mg·kg^−1^), and (5) oeERα‐A549 cells with fulvestrant (5 mg·kg^−1^). The drugs were administered every other day. The mice were sacrificed 4 weeks after drug treatment. Tumors were weighed, and IHC was performed.

**Fig. 8:**
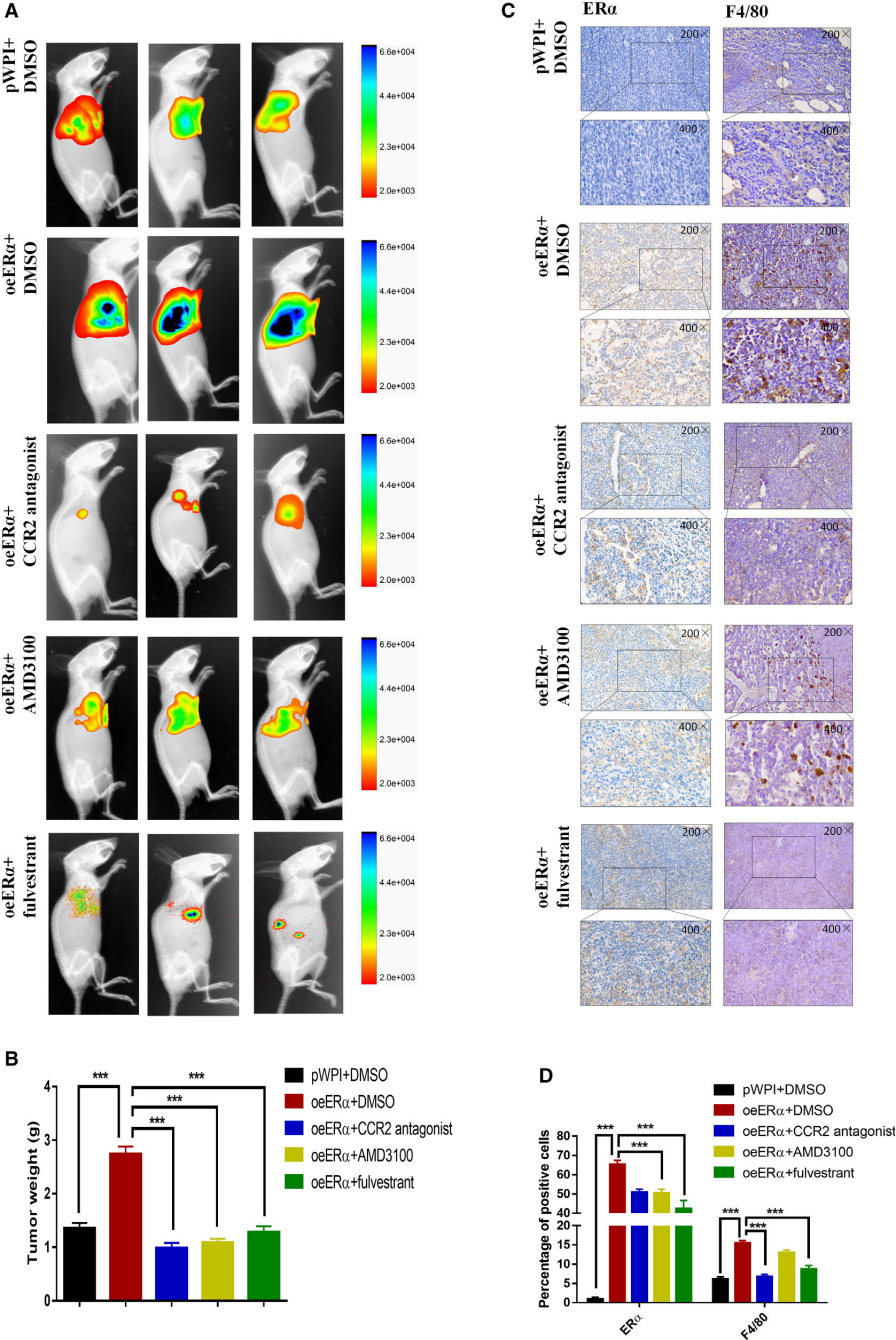
Estrogen receptor α promotes lung cancer progression using the *in vivo* mouse model. (A) Representative IVIS imaging for mice with A549‐Luc cells, with or without oeERα, after the respective treatments. (B) After 4 weeks of treatments, the mice were sacrificed and tumor weights were measured. (C,D) The IHC staining (C), and quantification (D) for detection of ERα and F4/80 expressions in different groups. 200× magnification was shown for the upper image, and 400× magnification was shown for the lower image for the IHC results. Student’s *t*‐test was used to analyze data in B,D. Experiments were done at least in 3 replicates. Results were presented as mean ± SD, ***P < 0.001.

As shown by the increased IVIS luminescence detection, overexpression of ERα in A549 cells can increase the tumor growth, which can be reduced by CCR2 antagonist, CXCR4 antagonist AMD3100, or anti‐estrogen fulvestrant *in vivo* (Fig. 8A). After mice were sacrificed, the tumor weight measurements were consistent with the IVIS results (Fig. 8B).

IHC with antibody against F4/80 was performed to confirm the induction of macrophage infiltration, which can be promoted by up‐regulation of ERα expression in lung cancer cells, and can partly be blocked by treatment with CCR2 antagonist or fulvestrant. Our data also showed the reduction of ERα expression by treating the mice with CCR2 antagonist, CXCR4 antagonist (AMD3100), or fulvestrant (Fig. 8C,D).

Together, results from Fig. 8A–D of mouse lung tumor model studies confirmed our *in vitro* results showing that ERα can play a positive role in promoting NSCLC progression via altering the CCR2‐ and CXCR4‐involved pathways.

## 4 Discussion

**Fig. 9:**
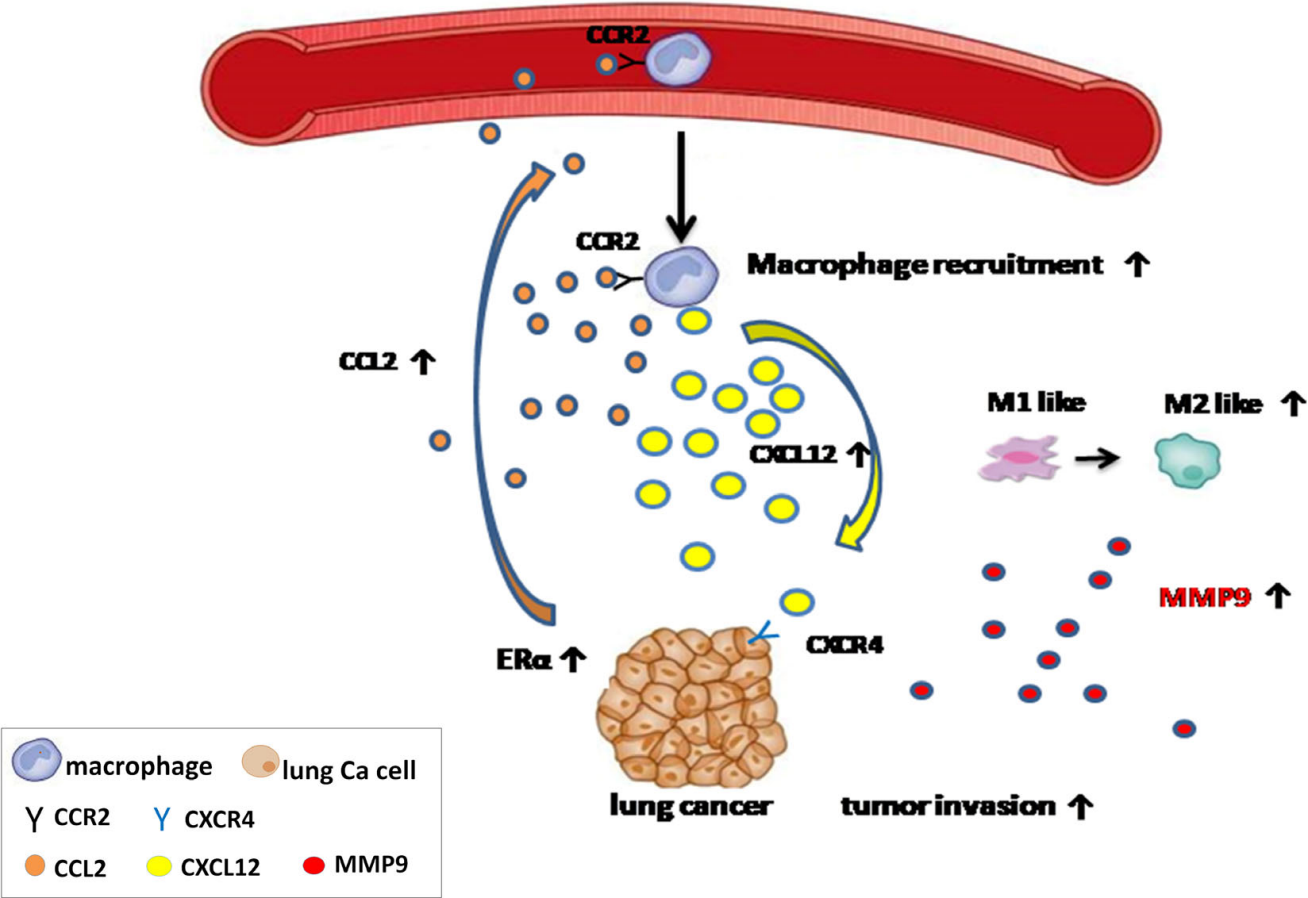
Schematic illustration of the cross‐talk between lung cancer cells and macrophages showing that (i) a higher ERα activity in lung cancer cells can promote the infiltration of macrophage via an increased CCL2 secretion, which can secrete MMP9 to promote lung cancer cell invasion; (ii) the infiltrated macrophages can secrete CXCL12 to bind to lung cancer cell surface gene CXCR4 to increase ERα expression.

In the present study, we proved that ERα expression in lung cancer cells can promote NSCLC invasion through increase of and cross‐talk with infiltrated macrophages via up‐regulating the CCL2‐ and CXCL12‐involved signal pathways. On the one hand, higher expression of ERα in lung cancer cells can lead to more production of CCL2, which can then bind to CCR2 receptor on the membrane of the infiltrated macrophages. This was also accompanied by higher macrophage infiltration, a better M2‐type macrophage polarization, and a higher MMP9 secretion from macrophages. The increased macrophage infiltration and higher MMP9 productions constitute a tumor microenvironment for promoting the lung cancer cell invasion. On the other hand, infiltrated macrophages could further influence the expression of ERα in lung cancer cells via a positive feedback mechanism. More infiltrated macrophages would produce a higher amount of CXCL12, which will act on the CXCR4 receptor on lung cancer cells and lead to activation of p‐ERK1/2 and p‐Akt and a consequent increase of ERα expression in lung cancer cells (Fig. 9).

In the past two decades, there have been several reports on the roles of ERα in NSCLC progression (Albain *et al*., 1991; Brueckl *et al*., 2013; Ganti *et al*., 2006; Kawai *et al*., 2005; Moore *et al*., 2003; Raso *et al*., 2009). While the majority of clinical evidence pointed to a tumor‐promoting role of ERα (Albain *et al*., 1991; Ganti *et al*., 2006; Kawai *et al*., 2005), ERα was also reported to have a protecting effect for cancer progression (Brueckl *et al*., 2013). However, the report with the conclusion of ERα protecting role was mainly based on analysis results from later stages of clinical samples, which may require further validation. In addition, the biphasic roles of ERα were reported during breast cancer progression. Though commonly regarded as a tumor‐promoting factor, ERα was found to inhibit breast cancer invasion (Gao *et al*., 2017; Padilla‐Rodriguez *et al*., 2018; Plate t *et al*., 2004). In the present study, our analysis of the online TCGA database on NSCLC samples from earlier stages (TNM stage IA‐IIB) shows significant tumor‐promoting effects of ERα in both LUAD and LUSC. TCGA data analyses based on different genders also reveal that the ERα tumor‐promoting effects were observed in male as well as in female NSCLC patients. The above data provide strong support for our hypothesis that ERα is a driving factor for the initiation and progression of NSCLC patients prior to the late stage.

Tumor‐associated macrophages have been shown to promote cancer progression (Noy and Pollard, 2014). While induction of ERα in lung cancer cells did not seem to directly induce tumor growth or invasion, immunofluorescence staining of human clinical samples showed that ERα in NSCLC samples is associated with higher staining levels of macrophage markers. This pointed to the linkage of ERα and infiltrating macrophages in NSCLC microenvironment. Furthermore, preclinical studies from NSCLC animal models show that lung cancer cells can induce the macrophage infiltration through increasing CCL2 and CXCL3 production of NSCLC cells (Schmall *et al*., 2015). Our results show that CCL2 is directly regulated by ERα, and the increased CCL2 in lung cancer cells can further influence the M2 polarization and MMP9 production of macrophages. Changing the CCL2 signals by shRNA or CCR2 antagonist can significantly reverse ERα’s induction of THP‐1 infiltration (Fig. 4E,G), MMP9 production (Fig. 4I,J), and invasion‐promoting effect of CM from coculture with THP‐1 (Fig. 4K,L). Previous evidence from breast cancer studies shows that CCL2 can be downstream of ERα to interact with macrophages (Svensson *et al*., 2015), and we further prove that the regulation is through a transcriptional regulation of the CCL2 promoter. It should also be mentioned that mRNA expression of IL‐10, which is a strong inhibitory cytokine, is also changed in correlation with that of ERα; thus, we do not rule out other paracrine pathways that may also be involved in the NSCLC–macrophage interaction induced by ERα.

Macrophages can secrete various factors to promote cancer cell invasion, and M2‐type polarization is generally associated with greater invasion‐promoting ability of macrophages (Shapouri‐Moghaddam *et al*., 2018). Thus, apart from the direct effect on the infiltration of macrophages, it is necessary to study the change of M2‐type polarization of macrophages after coculture with lung cancer cells with high or low expression of ERα. While the activation of CCR2 by CCL2 has been mainly shown to influence the recruitment of macrophages, recent evidence also revealed its effect on macrophage phenotype changes, including the secretion of MMPs (Arendt *et al*., 2013; Chen *et al*., 2018; Kersten *et al*., 2017; Okuma *et al*., 2004). Indeed, our results show that blocking the ERα/CCL2 axis by shCCL2 can reduce macrophage M2 polarization and MMP9 production, which will further lead to the change of invasion of lung cancer cells.

In addition to ERα’s effects on promoting the macrophage infiltration and function, previous reported data also suggested that infiltrated macrophages may influence the ERα expression in cancer cells. While macrophages can reduce ERα expression in breast cancer cells (Stossi *et al*., 2012), an induction effect was seen in endometrial cancer cells (Ning *et al*., 2016). It is possible that the macrophages‐regulated ERα expression can be different in different cancer scenarios. Our studies delineated the underlying mechanism by which macrophages up‐regulate ERα expression in lung cancer cells. Moreover, through the lentiviral transduction of two different shRNAs (shERα#1 and shERα#2) or the treatment with a selective ERα antagonist, we proved that the regulatory pathway is through the production of macrophage CXCL12 to activate its receptor, CXCR4, on lung cancer cells. While previous literature proved the interaction between CXCL12 and estrogen receptor (Sauvé *et al*., 2009), the detailed mechanisms were not depicted. The effect can also be partly diminished by inhibiting the downstream p‐ERK1/2 and p‐Akt of CXCR4, which is consistent with previous literature showing that ERK and Akt activations could lead to the induction of ERα transcription (Li *et al*., 2010; Stoica *et al*., 2003).

Compared with breast and endometrial cancer cells, the percentage of ERα‐positive cells in NSCLC tumor is relatively low. However, through the feedback interaction with infiltrating macrophages, it is possible that this vicious circle can lead to significant clinical effects. Moreover, our *in vitro* results, together with previous literature (Ganti *et al*., 2006), prove that the ERα expressed in lung cancer cells can respond to E2 activation, which provides further evidence that the ERα in lung cancer cells functions well.

## 5 Conclusions

While surgery, chemotherapy, and recently developed targeted therapy have been the major therapeutic methods used in the clinic, the development of novel therapies targeting various facets of pathogenesis is still needed to improve the overall survival of patients. Our study proved that ERα in lung cancer cells can promote the signal cross‐talk between lung cancer cells and macrophages via the ERα/CCL2/MMP9 and CXCL12/CXCR4 pathways. This interaction can lead to increased infiltration, M2‐type polarization, MMP9 production of macrophages, and a feedback induction of ERα expression of lung cancer cells (Fig. 8). The above changes in lung cancer tumor microenvironment could further lead to greater invasion of the lung cancer cells. NSCLC has been a great health issue worldwide, with high levels of incidence and mortality. Together, the interactions between lung cancer cells and macrophages through ERα/CCL2/CCR2/MMP9 and CXCL12/CXCR4 pathways could promote the NSCLC progression. The development of alternative new therapies by targeting ERα, CCR2, or CXCR4 may provide benefits for NSCLC patients in the future.

## Conflict of interest

The authors declare no conflict of interest.

## Authors contributions

MH designed and executed experiments and wrote the manuscript. XL and MH contributed to human specimen collection and pathological diagnoses. WY helped construct plasmids used for lentiviral infection. CC helped with experiment design and provided intellectual input. KJ and SY conceived the study, designed the experiments, and edited and approved the final version of the manuscript.

## Supporting information


**Fig S1.** ERα protein expression is correlated with worse prognosis in early‐stage NSCLC patients.

**Fig S2.** Immunofluorescence assays of ERα in A549 (Ctrl or oeERα), H1299 and LLC1 cells.

**Fig S3.** ERα/E2 effects on lung cancer cell growth and invasion.

**Fig S4.** Representative Western Blot images of detecting ERα, MMP9 and CXCL12 expressions and changes in lung cancer or macrophage cells.

**Table S1.** List of primer sequences used for qRT‐PCR.
**Table S2** **.** Characteristics of lung cancer patients from TCGA database.
**Table S3** **.** Characteristics of lung cancer patients from Wuhan Union Hospital.

## References

[mol212701-bib-0001] Albain KS , Crowley JJ , LeBlanc M and Livingston RB (1991) Survival determinants in extensive‐stage non‐small‐cell lung cancer: the Southwest Oncology Group experience. J Clin Oncol 9, 1618–1626. 1651993 10.1200/JCO.1991.9.9.1618

[mol212701-bib-0002] Ao J‐Y , Zhu X‐D , Chai Z‐T , Cai H , Zhang Y‐Y , Zhang K‐Z , Kong L‐Q , Zhang N , Ye B‐G , Ma D‐N *et al* (2017) Colony‐stimulating factor 1 receptor blockade inhibits tumor growth by altering the polarization of tumor‐associated Macrophages in Hepatocellular Carcinoma. Mol Cancer Ther 16, 1544–1554. 28572167 10.1158/1535-7163.MCT-16-0866

[mol212701-bib-0003] Arendt LM , McCready J , Keller PJ , Baker DD , Naber SP , Seewaldt V and Kuperwasser C (2013) Obesity promotes breast cancer by CCL2‐mediated macrophage recruitment and angiogenesis. Cancer Res 73, 6080–6093. 23959857 10.1158/0008-5472.CAN-13-0926 PMC3824388

[mol212701-bib-0004] Bröker LE , Huisman C , Span SW , Rodriguez JA , Kruyt FAE and Giaccone G (2004) Cathepsin B Mediates caspase‐independent cell death induced by microtubule stabilizing agents in non‐small cell lung cancer cells. Can Res 64, 27–30. 10.1158/0008-5472.can-03-3060 14729603

[mol212701-bib-0005] Brueckl WM , Al‐Batran SE , Ficker JH , Claas S , Atmaca A , Hartmann A , Rieker RJ and Wirtz RM (2013) Prognostic and predictive value of estrogen receptor 1 expression in completely resected non‐small cell lung cancer. Int J Cancer 133, 1825–1831. 23580323 10.1002/ijc.28209

[mol212701-bib-0006] Busonero C , Leone S , Bartoloni S and Acconcia F (2018) Strategies to degrade estrogen receptor alpha in primary and ESR1 mutant‐expressing metastatic breast cancer. Mol Cell Endocrinol 480, 107–121. 30389467 10.1016/j.mce.2018.10.020

[mol212701-bib-0007] Chen C , He W , Huang J , Wang B , Li H , Cai Q , Su F , Bi J , Liu H , Zhang B *et al* (2018) LNMAT1 promotes lymphatic metastasis of bladder cancer via CCL2 dependent macrophage recruitment. Nat Commun 9, 3826. 30237493 10.1038/s41467-018-06152-x PMC6148066

[mol212701-bib-0008] Chen M , Hsu I , Wolfe A , Radovick S , Huang K , Yu S , Chang C , Messing EM and Yeh S (2009) Defects of prostate development and reproductive system in the estrogen receptor‐α null male mice. Endocrinology 150, 251–259. 18755802 10.1210/en.2008-0044 PMC5398428

[mol212701-bib-0009] Cherney RJ , Mo R , Meyer DT , Nelson DJ , Lo YC , Yang G , Scherle PA , Mandlekar S , Wasserman ZR , Jezak H *et al* (2008) Discovery of disubstituted cyclohexanes as a new class of CC chemokine receptor 2 antagonists. J Med Chem 51, 721–724. 18232650 10.1021/jm701488f

[mol212701-bib-0010] Ding X , Yang D‐R , Xia L , Chen B , Yu S , Niu Y , Wang M , Li G and Chang C (2015) Targeting TR4 nuclear receptor suppresses prostate cancer invasion via reduction of infiltrating macrophages with alteration of the TIMP‐1/MMP2/MMP9 signals. Mol Cancer 14, 16. 25623427 10.1186/s12943-014-0281-1 PMC4316804

[mol212701-bib-0011] Farmaki E , Chatzistamou I , Kaza V and Kiaris H (2016) A CCL8 gradient drives breast cancer cell dissemination. Oncogene 35, 6309–6318. 27181207 10.1038/onc.2016.161 PMC5112152

[mol212701-bib-0012] Ganti AK , Sahmoun AE , Panwalkar AW , Tendulkar KK and Potti A (2006) Hormone replacement therapy is associated with decreased survival in women with lung cancer. J Clin Oncol 24, 59–63. 16314616 10.1200/JCO.2005.02.9827

[mol212701-bib-0013] Gao Y , Wang Z , Hao Q , Li W , Xu Y , Zhang J , Zhang W , Wang S , Liu S , Li M *et al* (2017) Loss of ERα induces amoeboid‐like migration of breast cancer cells by downregulating vinculin. Nat Commun 8, 14483. 28266545 10.1038/ncomms14483 PMC5344302

[mol212701-bib-0014] Harrington WR , Sheng S , Barnett DH , Petz LN , Katzenellenbogen JA and Katzenellenbogen BS (2003) Activities of estrogen receptor alpha‐ and beta‐selective ligands at diverse estrogen responsive gene sites mediating transactivation or transrepression. Mol Cell Endocrinol 206, 13–22. 12943986 10.1016/s0303-7207(03)00255-7

[mol212701-bib-0015] Hatse S , Princen K , Bridger G , De Clercq E and Schols D (2002) Chemokine receptor inhibition by AMD3100 is strictly confined to CXCR4. FEBS Lett 527, 255–262. 12220670 10.1016/s0014-5793(02)03143-5

[mol212701-bib-0016] Izumi K , Fang LY , Mizokami A , Namiki M , Li L , Lin WJ and Chang C (2013) Targeting the androgen receptor with siRNA promotes prostate cancer metastasis through enhanced macrophage recruitment via CCL2/CCR2‐induced STAT3 activation. EMBO Mol Med 5, 1383–1401. 23982944 10.1002/emmm.201202367 PMC3799493

[mol212701-bib-0017] Joyce JA and Pollard JW (2009) Microenvironmental regulation of metastasis. Nat Rev Cancer 9, 239–252. 19279573 10.1038/nrc2618 PMC3251309

[mol212701-bib-0018] Katz LH , Li Y , Chen J‐S , Muñoz NM , Majumdar A , Chen J and Mishra L (2013) Targeting TGF‐β signaling in cancer. Expert Opinion on Therapeutic Targets 17, 743–760. 23651053 10.1517/14728222.2013.782287 PMC3745214

[mol212701-bib-0019] Kawai H , Ishii A , Washiya K , Konno T , Kon H , Yamaya C , Ono I , Minamiya Y and Ogawa J (2005) Estrogen receptor alpha and beta are prognostic factors in non‐small cell lung cancer. Clin Cancer Res 11, 5084–5089. 16033821 10.1158/1078-0432.CCR-05-0200

[mol212701-bib-0020] Kersten K , Coffelt SB , Hoogstraat M , Verstegen NJM , Vrijland K , Ciampricotti M , Doornebal CW , Hau CS , Wellenstein MD , Salvagno C *et al* (2017) Mammary tumor‐derived CCL2 enhances pro‐metastatic systemic inflammation through upregulation of IL1beta in tumor‐associated macrophages. Oncoimmunology 6, e1334744. 28919995 10.1080/2162402X.2017.1334744 PMC5593698

[mol212701-bib-0021] Kirma N , Luthra R , Jones J , Liu Y‐G , Nair HB , Mandava U and Tekmal RR (2004) Overexpression of the Colony‐Stimulating Factor (CSF‐1) and/or its receptor c‐fms in mammary glands of transgenic mice results in hyperplasia and tumor formation. Can Res 64, 4162–4170. 10.1158/0008-5472.CAN-03-2971 15205327

[mol212701-bib-0022] Lazennec G and Richmond A (2010) Chemokines and chemokine receptors: new insights into cancer‐related inflammation. Trends Mol Med 16, 133–144. 20163989 10.1016/j.molmed.2010.01.003 PMC2840699

[mol212701-bib-0023] Lee H‐W , Choi H‐J , Ha S‐J , Lee K‐T and Kwon Y‐G . 2013 Recruitment of monocytes/macrophages in different tumor microenvironments. Biochim et Biophys Acta – Rev Cancer 1835, 170–179. 10.1016/j.bbcan.2012.12.007 23287570

[mol212701-bib-0024] Levano KS , Jung EH and Kenny PA (2011) Breast cancer subtypes express distinct receptor repertoires for tumor‐associated macrophage derived cytokines. Biochem Biophys Res Comm 411, 107–110. 21712030 10.1016/j.bbrc.2011.06.102

[mol212701-bib-0025] Li R , Hebert JD , Lee TA , Xing H , Boussommier‐Calleja A , Hynes RO , Lauffenburger DA and Kamm RD (2017) Macrophage‐secreted TNFα and TGFβ1 influence migration speed and persistence of cancer cells in 3D tissue culture via independent pathways. Can Res 77, 279–290. 10.1158/0008-5472.CAN-16-0442 PMC5243269 27872091

[mol212701-bib-0026] Li X‐Y , Lu Y , Sun H‐Y , Wang J‐Q , Yang J , Zhang H‐J , Fan N‐G , Xu J , Jiang J‐J , Liu R‐Y *et al* (2010) G protein‐coupled receptor 48 upregulates estrogen receptor α expression via cAMP/PKA signaling in the male reproductive tract. Development 137, 151–157. 20023170 10.1242/dev.040659

[mol212701-bib-0027] Merchant N , Nagaraju GP , Rajitha B , Lammata S , Jella KK , Buchwald ZS , Lakka SS and Ali AN (2017) Matrix metalloproteinases: their functional role in lung cancer. Carcinogenesis 38, 766–780. 28637319 10.1093/carcin/bgx063

[mol212701-bib-0028] Moore KA , Mery CM , Jaklitsch MT , Estocin AP , Bueno R , Swanson SJ , Sugarbaker DJ and Lukanich JM (2003) Menopausal effects on presentation, treatment, and survival of women with non‐small cell lung cancer. Ann Thor Surg 76, 1789–1795. 10.1016/s0003-4975(03)01024-5 14667585

[mol212701-bib-0029] Murdoch C , Giannoudis A and Lewis CE (2004) Mechanisms regulating the recruitment of macrophages into hypoxic areas of tumors and other ischemic tissues. Blood 104, 2224–2234. 15231578 10.1182/blood-2004-03-1109

[mol212701-bib-0030] Murray PJ (2017) Macrophage Polarization. Annu Rev Physiol 79, 541–566. 27813830 10.1146/annurev-physiol-022516-034339

[mol212701-bib-0031] Nagarsheth N , Wicha MS and Zou W (2017) Chemokines in the cancer microenvironment and their relevance in cancer immunotherapy. Nat Rev Immunol 17, 559–572. 28555670 10.1038/nri.2017.49 PMC5731833

[mol212701-bib-0032] Ning C , Xie B , Zhang L , Li C , Shan W , Yang B , Luo X , Gu C , He Q , Jin H *et al* (2016) Infiltrating macrophages induce ERalpha expression through an IL17A‐mediated epigenetic mechanism to sensitize endometrial cancer cells to estrogen. Cancer Res 76, 1354–1366. 26744532 10.1158/0008-5472.CAN-15-1260

[mol212701-bib-0033] Noy R and Pollard Jeffrey W (2014) Tumor‐associated macrophages: from mechanisms to therapy. Immunity 41, 49–61. 25035953 10.1016/j.immuni.2014.06.010 PMC4137410

[mol212701-bib-0034] Okudela K , Mitsui H , Woo T , Arai H , Suzuki T , Matsumura M , Kojima Y , Umeda S , Tateishi Y , Masuda M *et al* (2016) Alterations in cathepsin L expression in lung cancers. Pathol Int 66, 386–392. 27327955 10.1111/pin.12424

[mol212701-bib-0035] Okuma T , Terasaki Y , Kaikita K , Kobayashi H , Kuziel WA , Kawasuji M and Takeya M (2004) C‐C chemokine receptor 2 (CCR2) deficiency improves bleomycin‐induced pulmonary fibrosis by attenuation of both macrophage infiltration and production of macrophage‐derived matrix metalloproteinases. J Pathol 204, 594–604. 15538737 10.1002/path.1667

[mol212701-bib-0036] Olivo‐Marston SE , Mechanic LE , Mollerup S , Bowman ED , Remaley AT , Forman MR , Skaug V , Zheng Y‐L , Haugen A and Harris CC (2010) Serum estrogen and tumor‐positive estrogen receptor‐alpha are strong prognostic classifiers of non‐small‐cell lung cancer survival in both men and women. Carcinogenesis 31, 1778–1786. 20729390 10.1093/carcin/bgq156 PMC2981456

[mol212701-bib-0037] Padilla‐Rodriguez M , Parker SS , Adams DG , Westerling T , Puleo JI , Watson AW , Hill SM , Noon M , Gaudin R , Aaron J *et al* (2018) The actin cytoskeletal architecture of estrogen receptor positive breast cancer cells suppresses invasion. Nat Commun 9, 2980. 30061623 10.1038/s41467-018-05367-2 PMC6065369

[mol212701-bib-0038] Perez G , Olivares IM , Rodriguez MG , Ceballos GM and Garcia Sanchez JR (2012) Arginase activity in patients with breast cancer: an analysis of plasma, tumors, and its relationship with the presence of the estrogen receptor. Oncol Res Treat 35, 570–574. 10.1159/000343005 23038227

[mol212701-bib-0039] Platet N , Cathiard AM , Gleizes M and Garcia M (2004) Estrogens and their receptors in breast cancer progression: a dual role in cancer proliferation and invasion. Crit Rev Oncol/Hematol 51, 55–67. 10.1016/j.critrevonc.2004.02.001 15207254

[mol212701-bib-0040] Qian BZ and Pollard JW (2010) Macrophage diversity enhances tumor progression and metastasis. Cell 141, 39–51. 20371344 10.1016/j.cell.2010.03.014 PMC4994190

[mol212701-bib-0041] Rades D , Setter C , Dahl O , Schild SE and Noack F (2012) The prognostic impact of tumor cell expression of estrogen receptor‐α, progesterone receptor, and androgen receptor in patients irradiated for nonsmall cell lung cancer. Cancer 118, 157–163. 21713768 10.1002/cncr.26282

[mol212701-bib-0042] Raso MG , Behrens C , Herynk MH , Liu S , Prudkin L , Ozburn NC , Woods DM , Tang X , Mehran RJ , Moran C *et al* (2009) Immunohistochemical expression of estrogen and progesterone receptors identifies a subset of NSCLCs and correlates with *EGFR* mutation. Clin Cancer Res 15, 5359–5368. 19706809 10.1158/1078-0432.CCR-09-0033 PMC2893045

[mol212701-bib-0043] Sakumoto R , Hayashi K‐G , Fujii S , Kanahara H , Hosoe M , Furusawa T and Kizaki K (2017) Possible roles of CC‐ and CXC‐chemokines in regulating bovine endometrial function during early pregnancy. Int J Mol Sci 18, 742. 10.3390/ijms18040742 PMC5412327 28362325

[mol212701-bib-0044] Sánchez‐Martín L , Estecha A , Samaniego R , Sánchez‐Ramón S , Vega MÁ and Sánchez‐Mateos P (2011) The chemokine CXCL12 regulates monocyte‐macrophage differentiation and RUNX3 expression. Blood 117, 88–97. 20930067 10.1182/blood-2009-12-258186

[mol212701-bib-0045] Sauvé K , Lepage J , Sanchez M , Heveker N and Tremblay A (2009) Positive feedback activation of estrogen receptors by the CXCL12‐CXCR4 pathway. Can Res 69, 5793–5800. 10.1158/0008-5472.CAN-08-4924 19584281

[mol212701-bib-0046] Schmall A , Al‐tamari HM , Herold S , Kampschulte M , Weigert A , Wietelmann A , Vipotnik N , Grimminger F , Seeger W , Pullamsetti SS *et al* (2015) Macrophage and cancer cell cross‐talk via CCR2 and CX3CR1 is a fundamental mechanism driving lung cancer. Am J Respir Crit Care Med 191, 437–447. 25536148 10.1164/rccm.201406-1137OC

[mol212701-bib-0047] Schweiger A , Staib A , Werle B , Kras̆ovec M , Lah TT , Ebert W , Turk V and Kos J (2000) Cysteine proteinase cathepsin H in tumours and sera of lung cancer patients: relation to prognosis and cigarette smoking. Br J Cancer 82, 782–788. 10732746 10.1054/bjoc.1999.0999 PMC2374398

[mol212701-bib-0048] Shapouri‐Moghaddam A , Mohammadian S , Vazini H , Taghadosi M , Esmaeili S‐A , Mardani F , Seifi B , Mohammadi A , Afshari JT and Sahebkar A (2018) Macrophage plasticity, polarization, and function in health and disease. J Cell Physiol 233, 6425–6440. 10.1002/jcp.26429 PMC13482560 29319160

[mol212701-bib-0049] Slatore CG , Chien JW , Au DH , Satia JA and White E (2010) Lung cancer and hormone replacement therapy: association in the vitamins and lifestyle study. J Clin Oncol 28, 1540–1546. 20159813 10.1200/JCO.2009.25.9739 PMC2849773

[mol212701-bib-0050] Stoica GE , Franke TF , Moroni M , Mueller S , Morgan E , Iann MC , Winder AD , Reiter R , Wellstein A , Martin MB *et al* (2003) Effect of estradiol on estrogen receptor‐α gene expression and activity can be modulated by the ErbB2/PI 3‐K/Akt pathway. Oncogene 22, 7998–8011. 12970748 10.1038/sj.onc.1206769

[mol212701-bib-0051] Stossi F , Madak‐Erdogan Z and Katzenellenbogen BS (2012) Macrophage‐elicited loss of estrogen receptor‐alpha in breast cancer cells via involvement of MAPK and c‐Jun at the ESR1 genomic locus. Oncogene 31, 1825–1834. 21860415 10.1038/onc.2011.370 PMC3223561

[mol212701-bib-0052] Su B , Zhao W , Shi B , Zhang Z , Yu X , Xie F , Guo Z , Zhang X , Liu J , Shen Q *et al* (2014) Let‐7d suppresses growth, metastasis, and tumor macrophage infiltration in renal cell carcinoma by targeting COL3A1 and CCL7. Mol Cancer 13, 206. 25193015 10.1186/1476-4598-13-206 PMC4168121

[mol212701-bib-0053] Svensson S , Abrahamsson A , Rodriguez GV , Olsson A‐K , Jensen L , Cao Y and Dabrosin C (2015) CCL2 and CCL5 are novel therapeutic targets for estrogen‐dependent breast cancer. Clin Cancer Res 21, 3794–3805. 25901081 10.1158/1078-0432.CCR-15-0204

[mol212701-bib-0054] Teicher BA and Fricker SP (2010) CXCL12 (SDF‐1)/CXCR4 pathway in cancer. Clin Cancer Res 16, 2927–2931. 20484021 10.1158/1078-0432.CCR-09-2329

[mol212701-bib-0055] Tong H , Ke JQ , Jiang FZ , Wang XJ , Wang FY , Li YR , Lu W and Wan XP (2016) Tumor‐associated macrophage‐derived CXCL8 could induce ERalpha suppression via HOXB13 in endometrial cancer. Cancer Lett 376, 127–136. 27018308 10.1016/j.canlet.2016.03.036

[mol212701-bib-0056] Wang J , Tian Y , Phillips KLE , Chiverton N , Haddock G , Bunning RA , Cross AK , Shapiro IM , Le Maitre CL and Risbud MV (2013) Tumor necrosis factor α– and interleukin‐1β–dependent induction of CCL3 expression by nucleus pulposus cells promotes macrophage migration through CCR1. Arthritis Rheum 65, 832–842. 23233369 10.1002/art.37819 PMC3582738

[mol212701-bib-0057] Yeh C‐R , Slavin S , Da J , Hsu I , Luo J , Xiao G‐Q , Ding J , Chou F‐J and Yeh S (2016) Estrogen receptor α in cancer associated fibroblasts suppresses prostate cancer invasion via reducing CCL5, IL6 and macrophage infiltration in the tumor microenvironment. Mol Cancer 15, 7. 26790618 10.1186/s12943-015-0488-9 PMC4721150

[mol212701-bib-0058] Yu W , Ding J , He M , Chen Y , Wang R , Han Z , Xing EZ , Zhang C and Yeh S (2018) Estrogen receptor beta promotes the vasculogenic mimicry (VM) and cell invasion via altering the lncRNA‐MALAT1/miR‐145‐5p/NEDD9 signals in lung cancer. Oncogene 38, 1225–1238. 30250297 10.1038/s41388-018-0463-1

[mol212701-bib-0059] Zhang L , Zhang S , Yao J , Lowery FJ , Zhang Q , Huang W‐C , Li P , Li M , Wang X , Zhang C *et al* (2015) Microenvironment‐induced PTEN loss by exosomal microRNA primes brain metastasis outgrowth. Nature 527, 100–114. 26479035 10.1038/nature15376 PMC4819404

