# Supplementary figures and images for: Estrogen receptor α promotes lung cancer cell invasion via increase of and cross‐talk with infiltrated macrophages through the CCL2/CCR2/MMP9 and CXCL12/CXCR4 signaling pathways
Source: Mol Oncol. 2020 Jun 28;14(8):1779–99. doi: 10.1002/1878-0261.12701 (PMC7400793; doi:10.1002/1878-0261.12701)

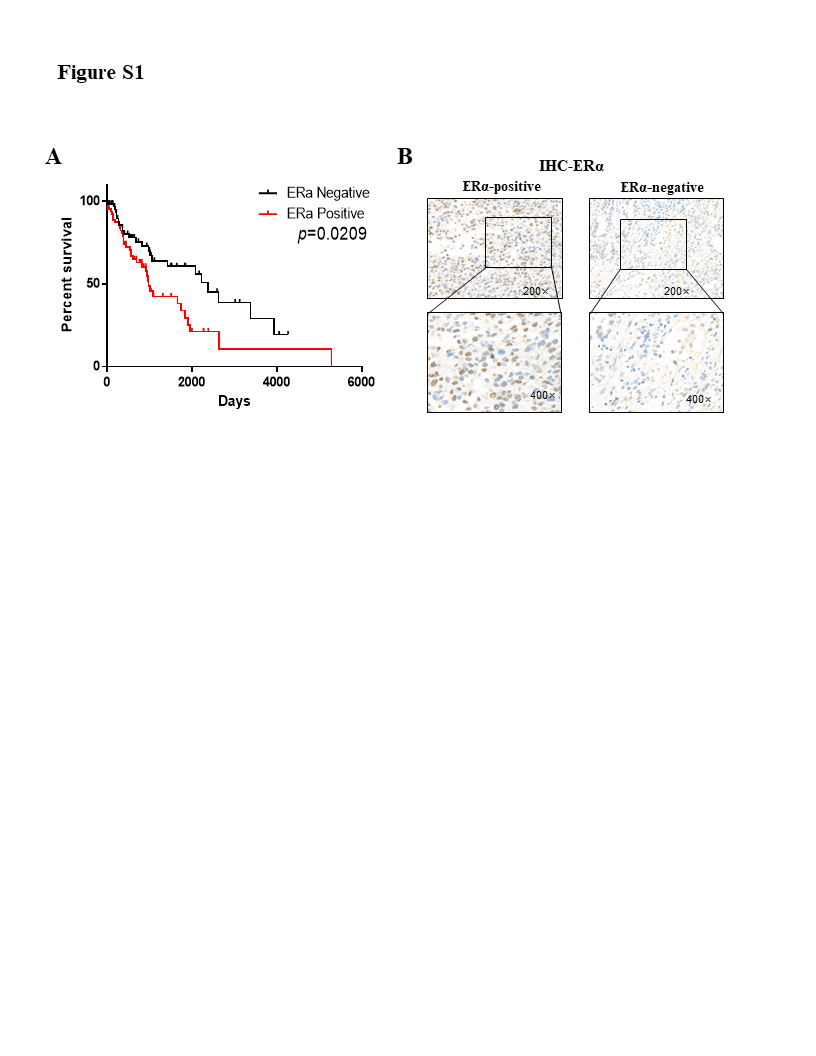

Supplement: Supplementary file 1 — Fig S1. ERα protein expression is correlated with worse prognosis in early‐stage NSCLC patients. [file MOL2-14-1779-s001.tif]

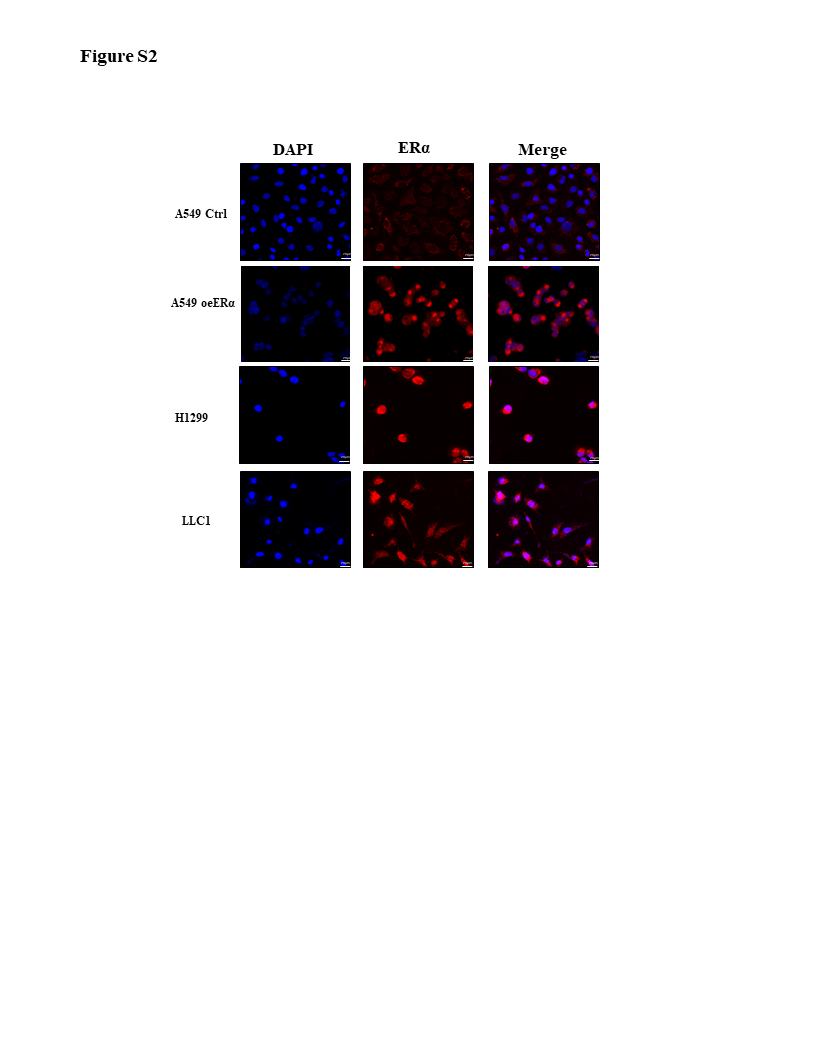

Supplement: Supplementary file 2 — Fig S2. Immunofluorescence assays of ERα in A549 (Ctrl or oeERα), H1299 and LLC1 cells. [file MOL2-14-1779-s002.tif]

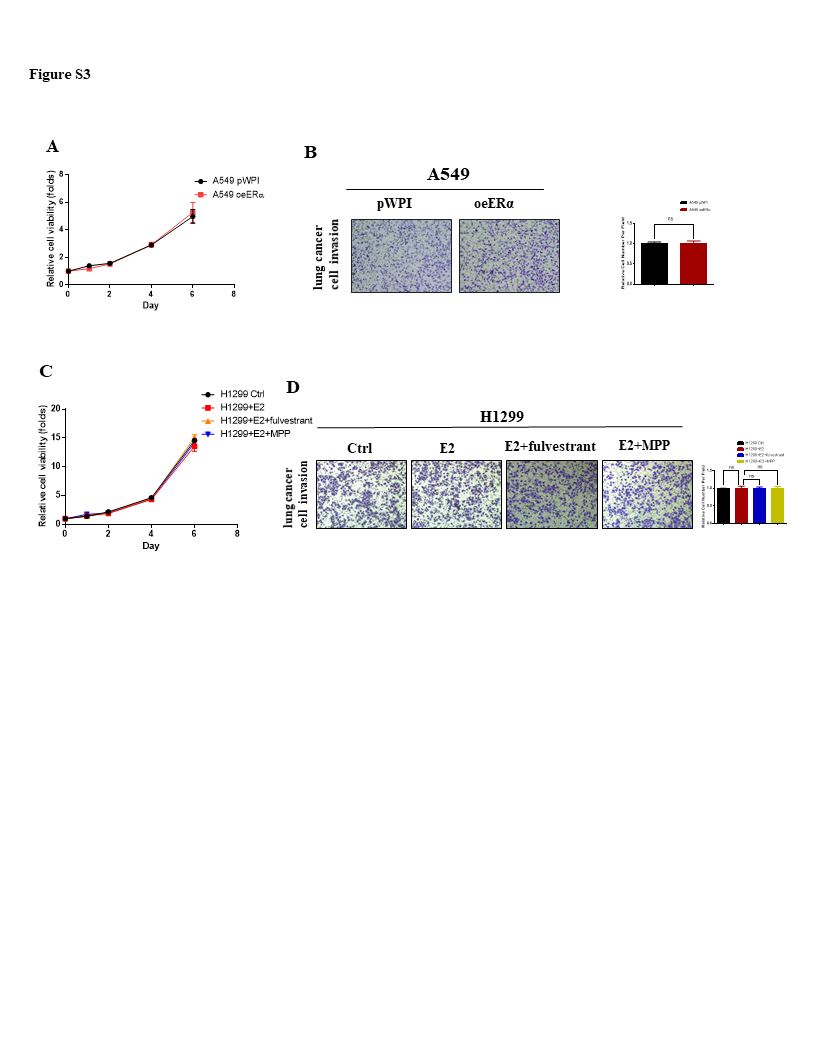

Supplement: Supplementary file 3 — Fig S3. ERα/E2 effects on lung cancer cell growth and invasion. [file MOL2-14-1779-s003.tif]

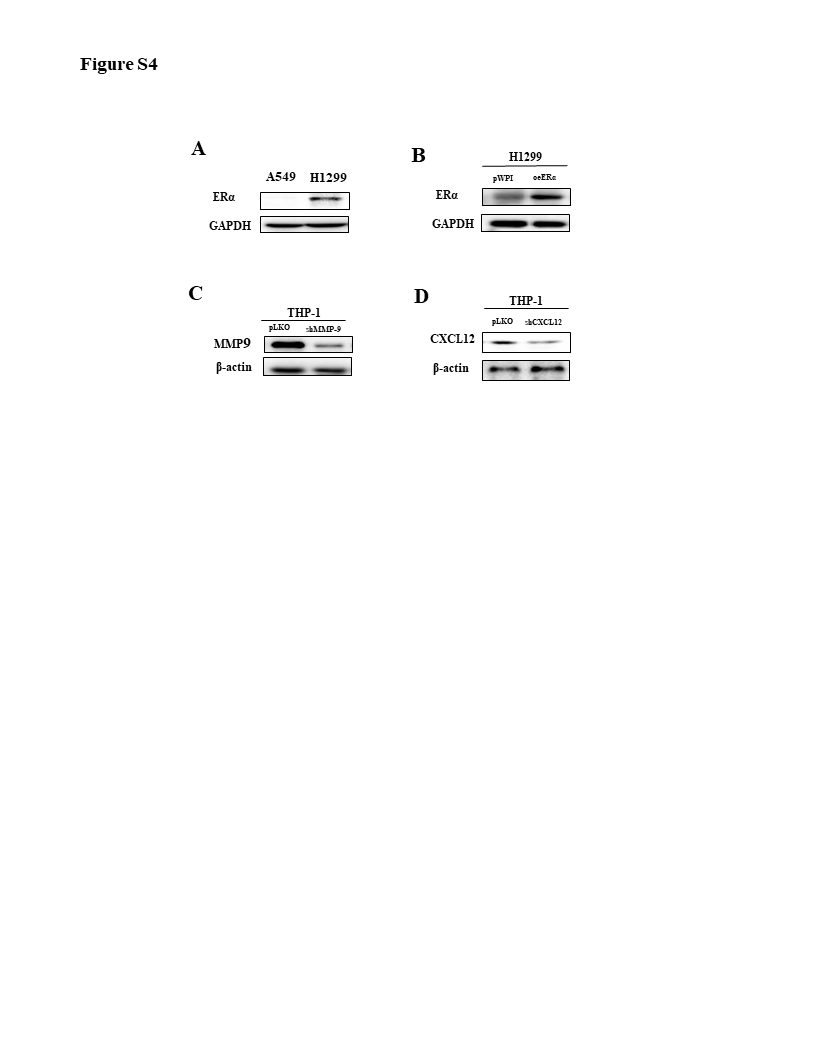

Supplement: Supplementary file 4 — Fig S4. Representative Western Blot images of detecting ERα, MMP9 and CXCL12 expressions and changes in lung cancer or macrophage cells. [file MOL2-14-1779-s004.tif]
