# Supplementary material for: Estrogen receptor α promotes lung cancer cell invasion via increase of and cross‐talk with infiltrated macrophages through the CCL2/CCR2/MMP9 and CXCL12/CXCR4 signaling pathways
Source: Mol Oncol. 2020 Jun 28;14(8):1779–99. doi: 10.1002/1878-0261.12701 (PMC7400793; doi:10.1002/1878-0261.12701)

**Table S1. Primer sequences for qPCR**

|  | **Forward** | **Reverse** | |
| --- | --- | --- | --- |
| **ERα** | CCCACTCAACAGCGTGTCTC | CGTCGATTATCTGAATTTGGCCT |  |
| **ARG1** | GTGGAAACTTGCATGGACAAC | AATCCTGGCACATCGGGAATC |  |
| **CD163** | TTTGTCAACTTGAGTCCCTTCAC | TCCCGCTACACTTGTTTTCAC |  |
| **CD206** | TCCGGGTGCTGTTCTCCTA | CCAGTCTGTTTTTGATGGCACT |  |
| **CCL22** | ATCGCCTACAGACTGCACTC | GACGGTAACGGACGTAATCAC |  |
| **CCR7** | TGAGGTCACGGACGATTACAT | GTAGGCCCACGAAACAAATGAT |  |
| **IL-6** | ACTCACCTCTTCAGAACGAATTG | CCATCTTTGGAAGGTTCAGGTTG |  |
| **β-actin** | CATGTACGTTGCTATCCAGGC | CTCCTTAATGTCACGCACGAT |  |
| **GAPDH** | GGAGCGAGATCCCTCCAAAAT | GGCTGTTGTCATACTTCTCATGG |  |
| **MMP1** | AAAATTACACGCCAGATTTGCC | GGTGTGACATTACTCCAGAGTTG |  |
| **MMP2** | TACAGGATCATTGGCTACACACC | GGTCACATCGCTCCAGACT |  |
| **MMP9** | TGTACCGCTATGGTTACACTCG | GGCAGGGACAGTTGCTTCT |  |
| **MMP14** | GGCTACAGCAATATGGCTACC | GATGGCCGCTGAGAGTGAC |  |
| **Cathepsin B** | GAGCTGGTCAACTATGTCAACA | GCTCATGTCCACGTTGTAGAAGT |  |
| **Cathepsin H** | CAAGTCATGGATGTCTAAGCACC | CATTGTTGTGGGCGTTTATCTTC |  |
| **Cathepsin L** | CTTTTGCCTGGGAATTGCCTC | CATCGCCTTCCACTTGGTC |  |
| **CCL2** | CAGCCAGATGCAATCAATGCC | TGGAATCCTGAACCCACTTCT |  |
| **CCL3** | AGTTCTCTGCATCACTTGCTG | CGGCTTCGCTTGGTTAGGAA |  |
| **CCL4** | CTGTGCTGATCCCAGTGAATC | TCAGTTCAGTTCCAGGTCATACA |  |
| **CCL5** | CCAGCAGTCGTCTTTGTCAC | CTCTGGGTTGGCACACACTT |  |
| **CCL7** | CTTCTGTGTCTGCTGCTCAC | GGGTCAGCACAGATCTCCTT |  |
| **CCL8** | TGGAGAGCTACACAAGAATCACC | TGGTCCAGATGCTTCATGGAA |  |
| **CX3CL1** | ACCACGGTGTGACGAAATG | TGTTGATAGTGGATGAGCAAAGC |  |
| **CXCL12** | ATTCTCAACACTCCAAACTGTGC | ACTTTAGCTTCGGGTCAATGC |  |
| **CSF-1** | TGGCGAGCAGGAGTATCAC | AGGTCTCCATCTGACTGTCAAT |  |
| **IL-1α** | TGGTAGTAGCAACCAACGGGA | ACTTTGATTGAGGGCGTCATTC |  |
| **IL-10** | GACTTTAAGGGTTACCTGGGTTG | TCACATGCGCCTTGATGTCTG |  |
| **IFN-γ** | TCGGTAACTGACTTGAATGTCCA | TCGCTTCCCTGTTTTAGCTGC |  |
| **TGF-β1** | GGCCAGATCCTGTCCAAGC | GTGGGTTTCCACCATTAGCAC |  |
| **TNF-α** | CCTCTCTCTAATCAGCCCTCTG | GAGGACCTGGGAGTAGATGAG |  |
| **IL-17** | TCCCACGAAATCCAGGATGC | GGATGTTCAGGTTGACCATCAC |  |
| **CXCL8** | CAGTTTTGCCAAGGAGTGCT | ACTTCTCCACAACCCTCTGC |  |
| **CXCL10** | GTGGCATTCAAGGAGTACCTC | TGATGGCCTTCGATTCTGGATT |  |
| **CCL18** | CCTGTGCACAAGTTGGTACC | ATTGGGGTCAGCACAGATCT |  |

**Table S2. Characteristics of patients from TCGA database**

| **Characteristics** | **Adenocarcinoma** | | | **Squamous cell carcinoma** | | |
| --- | --- | --- | --- | --- | --- | --- |
|  | **ERα low** | **ERα high** | **ERα low** | | **ERα high** |  |
| Number, n (%) | 190 (50.0%) | 190 (50.0%) | 198 (50.1%) | | 197 (49.9%) |  |
| Age, years (mean ± SD) | 65.31±10.19 | 65.86±9.26 | 66.78±8.43 | | 68.36±8.90 |  |
| Gender, n (%) |  |  |  | |  |  |
| Male | 99 (52.1%) | 72 (37.9%) | 150 (75.8%) | | 141 (71.6%) |  |
| Female | 91 (47.9%) | 118 (62.1%) | 48 (24.2%) | | 56 (28.4%) |  |
| Stage, n (%) |  |  |  | |  |  |
| I | 131 (68.9%) | 132 (69.5%) | 116 (58.6%) | | 123 (62.4%) |  |
| II | 59 (31.1%) | 58 (30.5%) | 82 (41.4%) | | 74 (37.6%) |  |

**Table S3. Characteristics of patients from Wuhan Union Hospital**

| **Characteristics** | **ERα negative** | **ERα positive** |
| --- | --- | --- |
| Number, n (%) | 64 (50.0%) | 64 (50.0%) |
| Age, years (mean ± SD) | 55.31 ± 22.04 | 54.19 ± 19.72 |
| Gender |  |  |
| Male | 44 (68.8%) | 34 (53.1%) |
| Female | 20 (31.2%) | 30 (46.9%) |
| Pathological type, n (%) |  |  |
| Adenocarcinoma | 28 (43.8%) | 30 (46.9%) |
| Squamous cell carcinoma | 36 (56.2%) | 34 (53.1%) |
| Stage, n (%) |  |  |
| I | 40 (62.5%) | 43 (67.2%) |
| II | 24 (37.5%) | 21 (32.8%) |


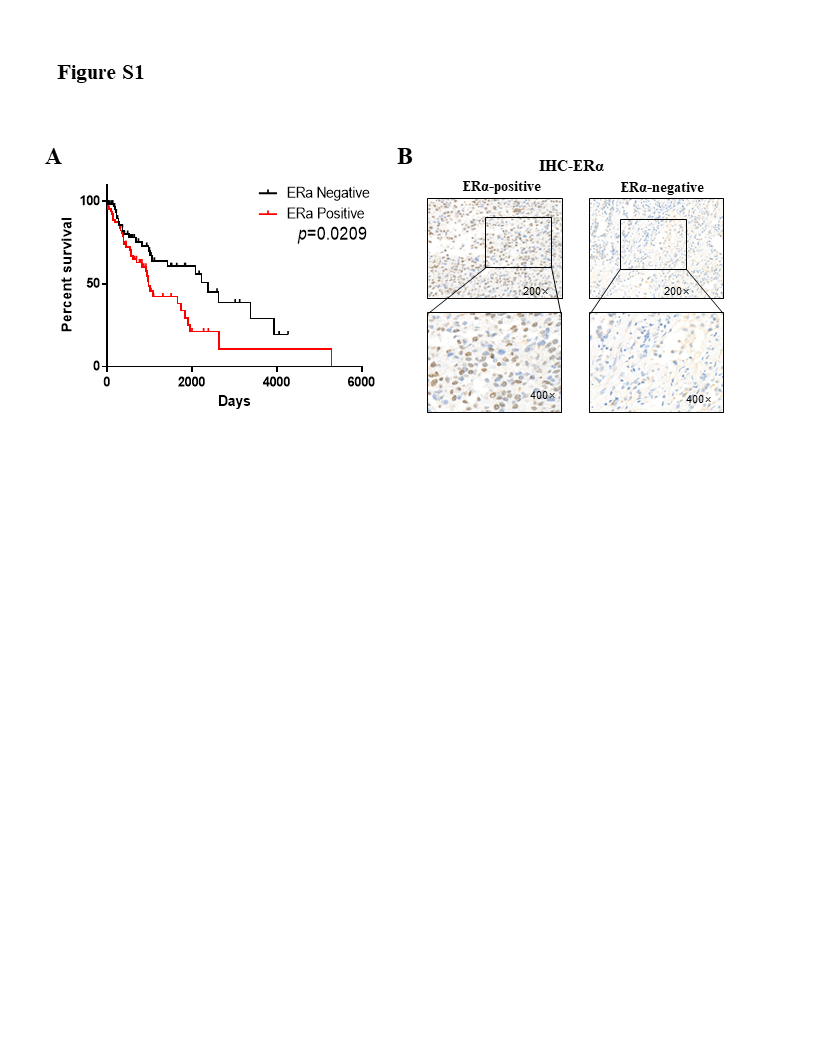


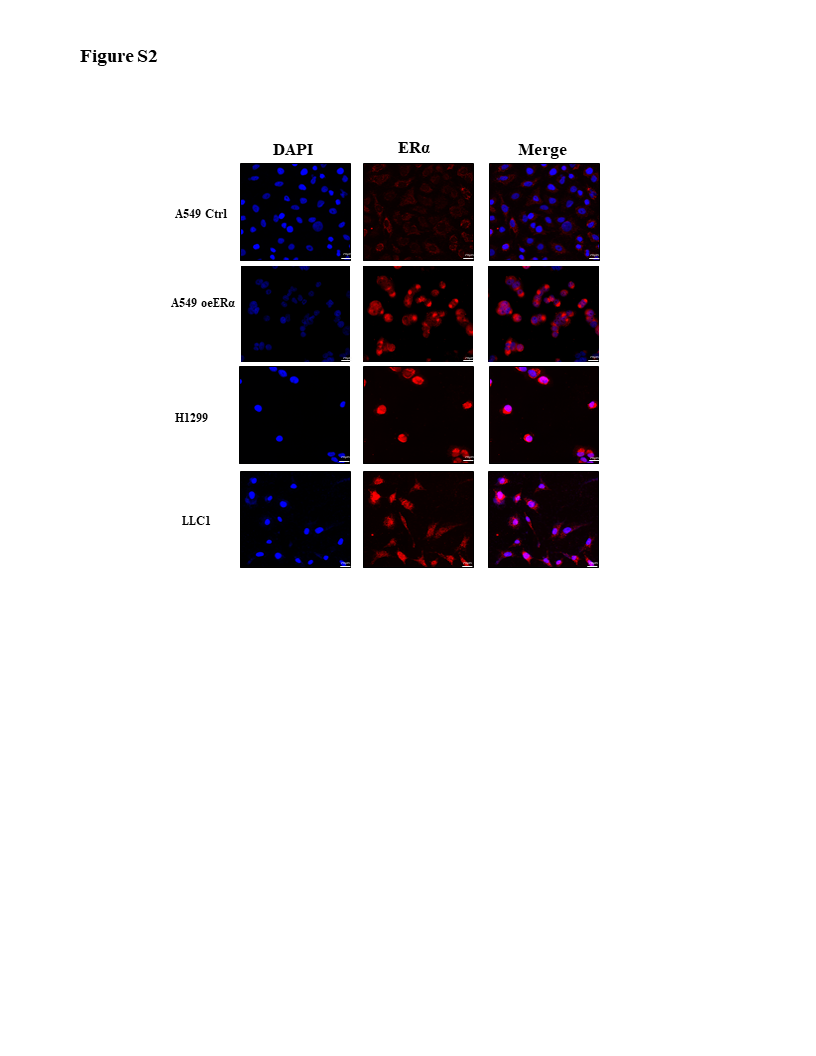


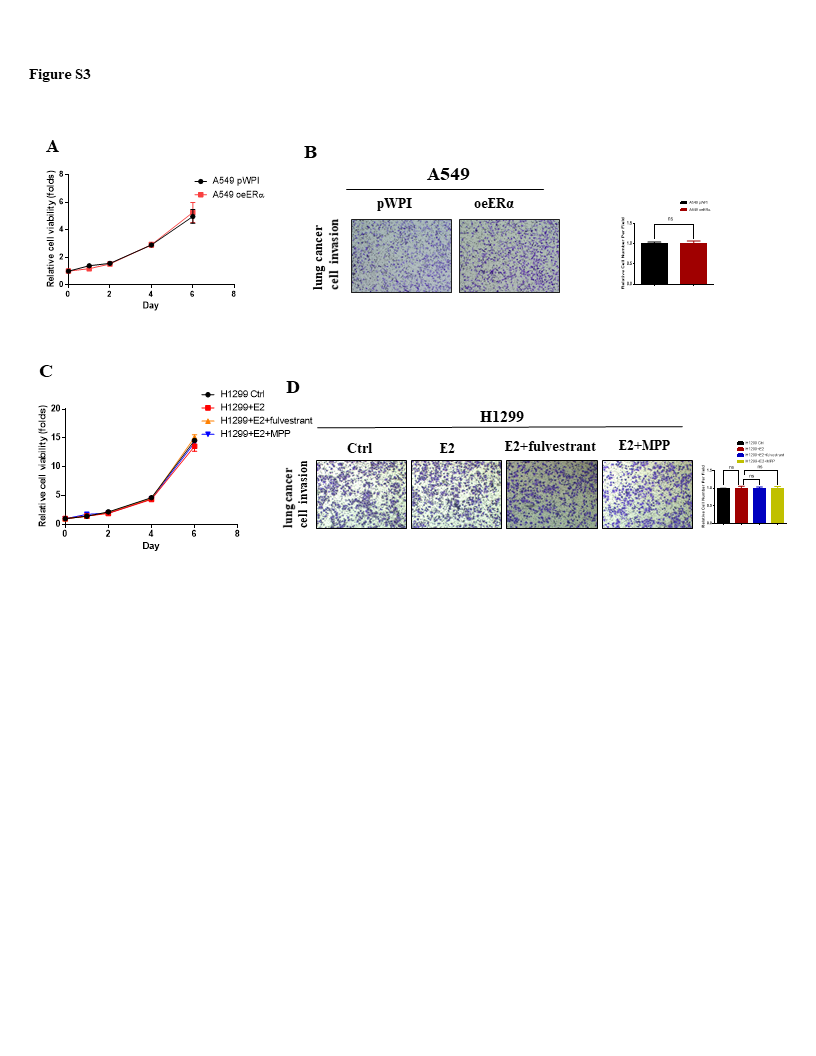


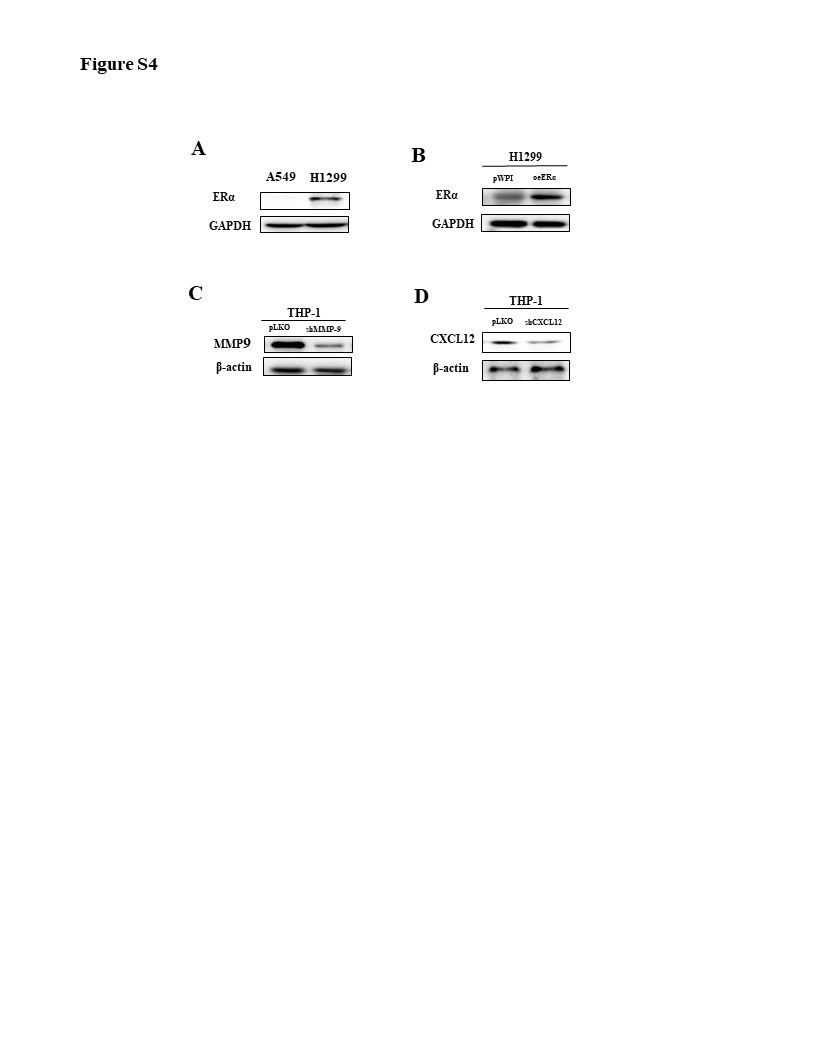

Supplement: Supplementary file 5 — Table S1. List of primer sequences used for qRT‐PCR. Table S2 . Characteristics of lung cancer patients from TCGA database. Table S3 . Characteristics of lung cancer patients from Wuhan Union Hospital. [file MOL2-14-1779-s005.docx]
